# A hybrid empirical and parametric approach for managing ecosystem complexity: Water quality in Lake Geneva under nonstationary futures

**DOI:** 10.1073/pnas.2102466119

**Published:** 2022-06-22

**Authors:** Ethan R. Deyle, Damien Bouffard, Victor Frossard, Robert Schwefel, John Melack, George Sugihara

**Affiliations:** ^a^Department of Biology, Boston University, Boston, MA 02215;; ^b^Scripps Institution of Oceanography, University of California San Diego, La Jolla, CA 92093;; ^c^Department of Surface Waters–Research and Management, Eawag–Swiss Federal Institute of Aquatic Science and Technology, Kastanienbaum, 6047, Switzerland;; ^d^Centre Alpin de Recherche sur les Réseaux Trophiques des Ecosystèmes Limniques, Univ. Savoie Mont Blanc, INRAE, Thonon-les-Bains, 73376, France;; ^e^Department of Ecology, Evolution, and Marine Biology, University of California, Santa Barbara, CA 93106;; ^f^Department of Ecohydrology and Biogeochemistry, Leibniz-Institute of Freshwater Ecology and Inland Fisheries, Berlin, 12587, Germany;; ^g^Bren School of Environmental Science and Management, University of California, Santa Barbara, CA 93106

**Keywords:** empirical dynamic modeling, water quality, reoligotrophication, aquatic ecosystem management, environmental data science

## Abstract

Severe deterioration of water quality in lakes, characterized by overabundance of algae and declining dissolved oxygen in the deep lake (DO_B_), was one of the ecological crises of the 20th century. Even with large reductions in phosphorus loading, termed “reoligotrophication,” DO_B_ and chlorophyll (CHL) have often not returned to their expected pre–20th-century levels. Concurrently, management of lake health has been confounded by possible consequences of climate change, particularly since the effects of climate are not neatly separable from the effects of eutrophication. Here, using Lake Geneva as an iconic example, we demonstrate a complementary alternative to parametric models for understanding and managing lake systems. This involves establishing an empirically-driven baseline that uses supervised machine learning to capture the changing interdependencies among biogeochemical variables and then combining the empirical model with a more conventional equation-based model of lake physics to predict DO_B_ over decadal time-scales. The hybrid model not only leads to substantially better forecasts, but also to a more actionable description of the emergent rates and processes (biogeochemical, ecological, etc.) that drive water quality. Notably, the hybrid model suggests that the impact of a moderate 3°C air temperature increase on water quality would be on the same order as the eutrophication of the previous century. The study provides a template and a practical path forward to cope with shifts in ecology to manage environmental systems for non-analogue futures.

## Significance

This paper develops a hybrid approach to account for the complex interactions affecting lake water quality and its management in a nonlinear, changing world. The approach uses data to leverage our first-principles understanding of the mechanisms operating on dissolved oxygen in the lake. This yields a manageable, but more complete systems perspective for environmental management of the lake under climate change, where our analysis suggests that multiple modes of intervention may be necessary to achieve a healthy lake.

During the 20th century, water quality in lakes across the globe declined as cities grew and agriculture industrialized (1). These declines were marked by high chlorophyll (CHL) from abundant algae and depressed dissolved oxygen levels in deep water (DO_B_) from excess organic matter (2). The prime culprit was enrichment of phosphorus (3). To counter and reverse eutrophication, remediation measures were implemented in many areas, such as construction of treatment plants that removed phosphorus and regulation of additives in laundry detergents. The logic was that by reducing phosphorus inputs (termed reoligotrophication), water quality would improve (4). However, contrary to expectation, even in systems with successful reoligotrophication, DO_B_ and CHL have often not returned to their earlier states.

Scientists have pursued many strategies to parametric modeling for lake systems (5–9), with a concerted effort to address the lack of success in water-quality improvement (10, 11). These approaches have favored different balances of complexity and resolution to address the interactions among total phosphorus (TP), CHL, and DO_B_ and how they operate within a nexus of interdependent lake physics, biogeochemistry, and ecology. However, the fundamentally complex nature of these interactions can make it difficult to uniquely identify and mathematically represent the causal pathways underlying system changes and management outcomes (12). Often, different combinations of rules and relationships can produce the same expectations and, hence, comparable fits to history. Ultimately, the question of “which” and “how many” components to include in a reductionistic description of these natural systems faces a fundamental trade-off between having too many parameters and too limited a set of relationships. Models with too many parameters lack predictive credibility due to overfitting (8), while models with too restricted a set of relationships are only credible for predicting behavior in specific system states (e.g., one particular primary producer community and associated biogeochemical rates).

The issue of overfitting notwithstanding, it is critical for the ongoing management of lake systems to have reliable models that can accommodate complexity and capture behavior across a range of states. Through the last century, eutrophication and reoligotrophication have caused broad ecological and biogeochemical changes (13–17) in the water column and sediments of many lakes. Additionally, a second major anthropogenic driver, climate change (18, 19), is beginning to exert strong influence on lake systems. Even just considering increasing air temperature,* there are manifold possible direct and indirect effects on lakes. Atmospheric warming is associated with changes in thermal structure and mixing regimes of many lakes (20). In deep temperate lakes specifically, increases in the strength and duration of stratification are expected to suppress winter mixing that resupplies oxygen to depth and nutrients to the surface (14, 21, 22). These physical changes can also affect water quality through biology, such as promoting less edible and harmful cyanobacteria (23). Thus, the consequences of reoligotrophication and atmospheric warming can be interrelated and synergistic. This begs the question: Is it possible to resolve the major interdependencies in the limnology without creating a model too complex to reliably fit or understand?

As a practical solution to this conundrum, we suggest a hybrid approach for modeling limnological complexity, which we demonstrate here using two-phase analysis of the iconic (24) case of Lake Geneva. First, we pursue a data-driven approach (25–29) with Empirical Dynamic Modeling (EDM) to identify interdependencies among causal drivers of DO_B_. Importantly, the empirical dynamic approach captures the net relationship among variables through time (25) and, thus, avoids the tricky business of distinguishing among specific, mechanistic rules (12). The causal analysis confirms that the incomplete recovery of the lake can be understood from documented changes in the ecology (particularly in the food web) that altered the cause-and-effect relationships with TP (13, 14, 18, 30) (*SI Appendix*, Fig. S1). Second, in the spirit of Mooij et al. (7), we investigate a hybrid-modeling approach, but instead of combining different parametric structures, here, we combine empirical (inductive) elements derived from data with parameterized (deductive) elements derived from first principles. It is designed with a modular structure, taking advantage of the reasonable assumption that biogeochemical processes in the lake do not feed back on atmospheric forcing. The oxygen sources to deep water from wind-driven mixing and river discharge are accounted for with a two-box model controlled by the one-dimensional (1D) hydrodynamic Simstrat model (21), while oxygen depletion in the deep lake is modeled with a multivariate empirical dynamic model we develop herein. However, the modularity means that the essential principle could easily be adjusted to a different hydrodynamic model or alternative nonlinear empirical dynamic predictor that might be more suitable in other applications.

The resulting hybrid model is a more complete systems perspective, which we validate by improved historical prediction (relative to current state-of-the-art). The hybrid model is then used to make iterative, multi-decadal calculations for Lake Geneva that explore water quality and hypoxic conditions under different reoligtrophication and air-temperature scenarios, demonstrating how the empirical–parametric approach can be a critical tool for adaptive management going forward, where multivariate complexity cannot be ignored.

## Reoligotrophication and State-Dependent Effects

**Fig. 1.**
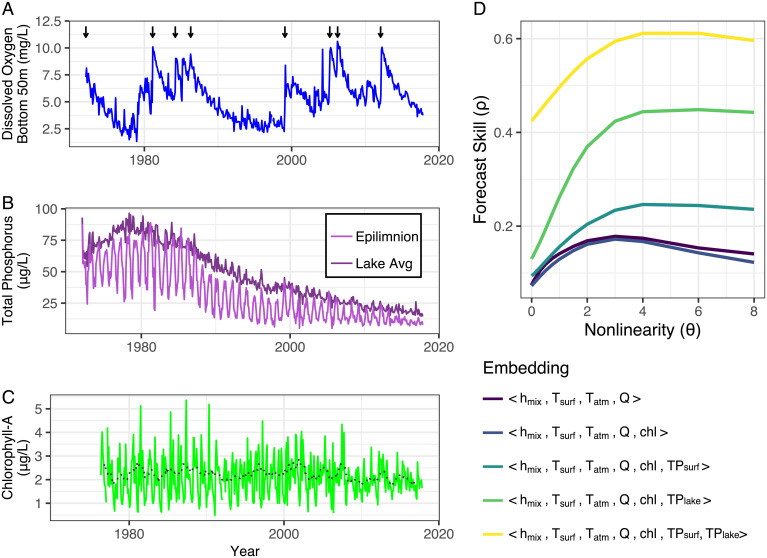
(*A*) Time series of DO_B_ averaged from 260 to 310 m in Lake Geneva. Vertical black arrows represent years in which Simstrat predicts mixing below 250 m. (*B*) Time series of lake TP both averaged over the entire lake and just the epilimnion (*Materials and Methods*). (*C*) Time series of averaged (0 to 30 m) of CHL-a. (*D*) Multivariate EDM analysis shows improvement in forecast skill (Pearson’s correlation between observed and predicted DO_B_) with sequential addition of biogeochemical variables to the embedding (set of coordinate variables). Variables are abbreviated as follows: h_mix_ = depth of mixed layer (epilimnion), T_surf_ = temperature of epilimnion, T_atm_ = air temperature, Q = Rhone River discharge, chl = CHL-a, TP_surf_ = concentration of TP averaged over the epilimnion, and TP_lake_ = concentration of TP averaged over the lake. The forecast skill is shown as a function of the nonlinear tuning parameter, θ. A MAR model (S-map with θ = 0) that does not allow for nonlinear state dependence between variables only reproduces part of the historical variance. The EDM approach is similar to multiple-linear-regression techniques, as it identifies the relationships among parameters. A fundamental difference is that EDM allows those relationships to change, depending on the state of the system.

In Lake Geneva, the dominant process driving the large-scale temporal evolution of DO_B_ is irregular deep mixing in winter (Fig. 1*A*). When surface cooling coincides with sufficiently strong wind, DO mixes from near-surface to deepest depths. However, atmospheric warming is predicted to interfere with this process. Schwefel et al. (21) modeled the effect of warming on the future water quality of Lake Geneva using a hydrodynamic model for thermal structure (Simstrat) coupled to a two-box model of oxygen with simple parameterizations for biogeochemical processes. The decreased frequency of deep mixing under warming was predicted to negatively affect DO_B_ more than previous eutrophication.

The study provides a good first approximation to lake water quality under future climate, but the structure of the model assumes that relationships between biological parameters are fixed and uses parameters that are only strictly valid for a limited range of system states. In particular, the model assumes a simple, fixed relationship between nutrients (TP) and algae abundance (CHL), but recent studies on the lake indicate the need to address biogeochemical interactions across a wider range of system-states. First, sediment cores show that Lake Geneva was not hypoxic prior to 1945 (31), when TP < 10 μg⋅L^−1^. Second, the phytoplankton communities in Lake Geneva and other Swiss lakes have shifted C:P stoichiometry to compensate for decreasing TP and, hence, increasing phosphorus limitation during early reoligotrophication (32, 33). The initial actions to lower TP had little effect on algal biomass and DO_B_ until phosphorus fell below 36 μg⋅L^−1^. Third, nonlinear causality analysis (30) found an evolving seasonal variability in the connectivity of biological, chemical, and physical variables during reoligotrophication, rather than stationary relationships.

While the general notion of complex interdependence is commonly accepted in limnology, an actionable quantitative understanding of the coupled effects of reoligotrophication and climate change remains an unfulfilled management goal. To construct a data-derived approach to this goal, we first establish an empirical foundation by performing a causality analysis for Lake Geneva similar to Anneville et al. (30), but that is explicitly focused on DO_B_ and a preselected subset of suspected ecological drivers (Fig. 1 *B* and *C* and *SI Appendix*, Fig. S2). For readers unfamiliar with EDM, we recommend two short videos (34, 35) for graphical description and narrated explanation of EDM basics. The nonlinear causal measurement method, convergent cross-mapping (CCM), can detect dynamic coupling among variables lacking fixed or well-defined correlations (26) [see short video (36)]. The basic causal drivers on DO_B_ found are air temperature, lake temperature, thermal structure, CHL, and phosphorus (*SI Appendix*, Table S1). Note that phosphorus is characterized with two depth averages (Fig. 1*B*): TP_surf_ (average TP in the top 20 m) tracks the seasonal cycling of phosphorus in and out of the euphotic zone, while TP_lake_ (average TP over the full lake hypsometry) tracks the long-term trend of the total mass of phosphorus in the system under reoligotrophication.

Causal coupling is further clarified with multivariate prediction (Fig. 1*D*). That is, we have greater confidence that a driver is important if it can contribute to prediction skill. However, the most familiar tool of multivariate prediction, linear regression, presupposes that the system is confined to a narrow range of behavior around an equilibrium, such that the dynamic relationships are fixed and can be treated by a single set of coefficients. When a natural system is changing through time, it is better to conceptualize the state not as jiggling around an equilibrium, but following an evolving trajectory on or near a dynamic attractor (again, we refer unfamiliar readers to the two short videos above for graphical explanation of attractor dynamics in the context of EDM). Linear multivariate models can approximate the dynamics, but the regression coefficients must be computed differently for each different “system state,” where each state is literally a specific location on the attractor defined by the particular values of the variables. This is exactly accomplished in EDM with multivariate S-map regression (37). S-maps approximate the local linear dynamics with weighted (kernel) regression, where each observation in the training set *X_i_* is weighted by using a decaying exponential of the distance (in state-space) to the state at time *t.* That is, $w_{i}=\text{exp}(-\theta d_{i}/\bar{d})$, where $d_{i}$ is the Euclidian distance to the target, and $\bar{d}$ is the average distance of observations in the training set. Defined this way, there is a parameter *θ* controlling the “steepness” of the weighting—i.e., the degree of local state-dependence. When *θ* = 0, all observations have equal weight, thus giving a standard global linear regression. When *θ* increases, the regression becomes increasingly sensitive to the observations closest to the target, and, hence, the regression is increasingly nonlinear. This is illustrated in *SI Appendix*, Fig. S3.

Here, we use an S-map to construct an additive comparison of multivariate DO_B_ prediction. Baseline predictability is set with a multivariate EDM model that uses the important physical drivers that go into the parametric Simstrat model and are validated by CCM (*SI Appendix*, Table S1): *h_mix_* = depth of the mixed layer (= depth of thermocline), *T_surf_* = temperature of the epilimnion, *T_atm_* = air temperature, and *Q* = Rhone River discharge. This four-dimensional empirical model is then augmented sequentially by using biological and biogeochemical variables identified by CCM. Indeed, adding biogeochemical terms improves prediction skill, and the best representation of the system (i.e., the attractor coordinates producing the best predictions) is obtained by integrating physical and biological information with both TP averages.

As described above, the degree of local state-dependence with the S-map is controlled by the nonlinear parameter *θ*. The linear S-map (*θ* = 0) is equivalent to a multivariate autoregressive (MAR) model and does not allow for state-dependence between variable effects. Fig. 1*D* shows that a global linear model only captures a small part of the dynamics (predictability) of DO_B_, and predictability improves substantially when the EDM models are tuned toward nonlinear solutions (*θ* > 0)—that is, the lake dynamics can be approximated by a limited set of variables (low-dimensional model), just not as linear dynamics around a stable equilibrium. This test validates the general hypothesis that interdependence among variables has shaped water-quality dynamics over the management history of Lake Geneva, but also shows that this complexity is quantitatively tractable (in the predictive sense).

**Fig. 2.**
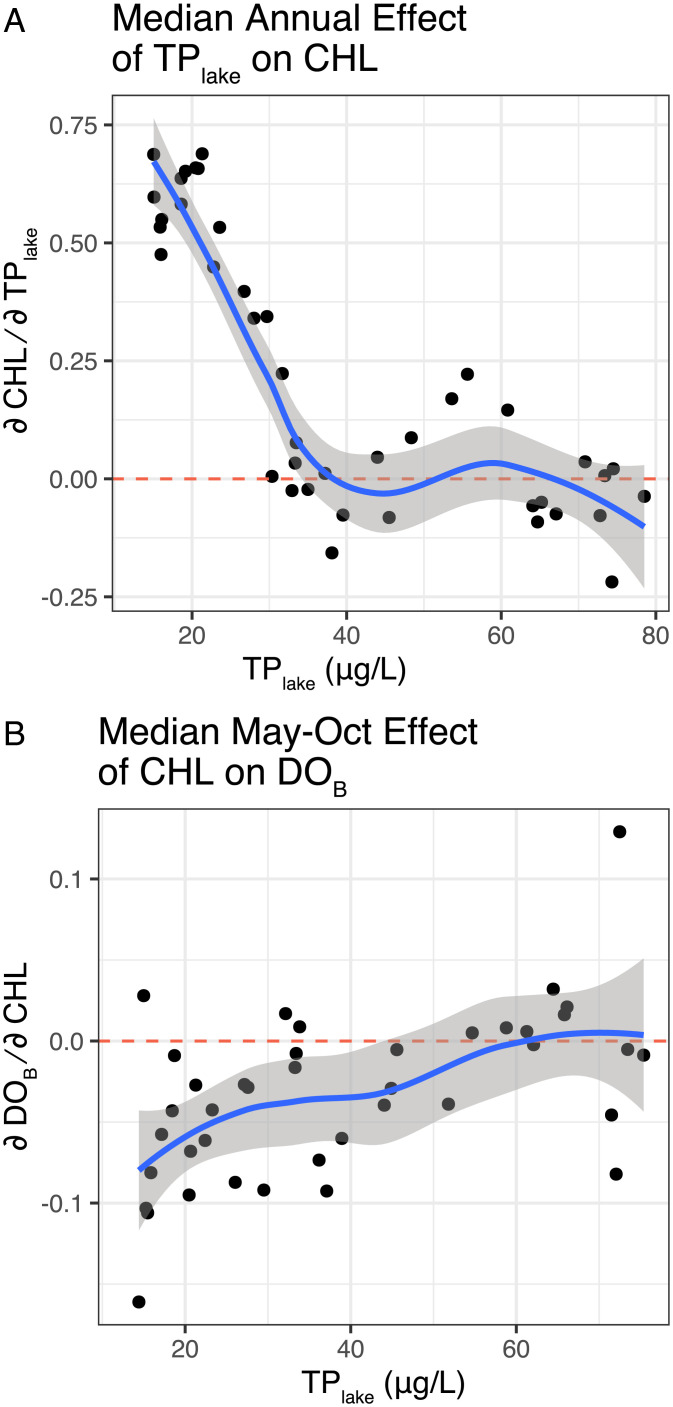
S-map estimates of first-order partial derivatives (Jacobian elements) quantify the state-dependent nature of interactions among phosphorus, CHL, and DO under reoligotrophication. (*A*) Effect of phosphorus on CHL. Positive values of ∂chl/∂TP indicate that an increase in TP_lake_ leads to an increase in CHL. At high levels of TP_lake_ (e.g., 1980s when TP_lake_ > 60 µg⋅L^−1^), the mean annual effect of TP_lake_ on CHL was essentially zero. There is little evidence of phosphorus ($\partial \text{CHL}/\partial {\text{TP}}_{\text{lake}}$ ∼ 0) until the TP_lake_ concentration drops below 40 µg⋅L^−1^. (*B*) S-map analysis finds the effect of CHL on DO changes with reoligotrophication (TP_lake_ concentration). Negative values of $\partial {\text{DO}}_{\text{B}}/\partial \text{CHL}$ show that, as expected, a decrease in CHL is usually associated with increased water quality (increased DO_B_). However, this is not always true, and at higher TP_lake_, the average effect of additional CHL is essentially zero.

Additional insight can be obtained from the fact that the local linear regression coefficients of the S-map quantify and track the changing strengths (and signs) of interactions between model variables through time (25). If the S-map includes all the key causal variables (a “mechanistic embedding”), the coefficients approximate the Jacobian partial derivatives between causal components (if key causal variables are missing, the direct interpretation of coefficients is less rigorous). In the context of management, we aim to quantify changes in the effect of TP on CHL and CHL on DO_B_. This first involves determining optimal multivariate S-map embeddings for CHL and DO_B_ (*Materials and Methods*). Then, the effect of TP_surf_ on CHL through time is quantified by the S-map coefficient estimating $\frac{\partial \mathrm{CHL}}{\partial TP}$ in the mechanistic embedding to predict CHL(*t* + 1) (*SI Appendix*, Fig. S5). In effect, this measures phosphorus limitation as a function of lake state. If production in the lake is TP limited, a small increase in TP will lead to an increase in CHL, and the coefficient will be positive. The empirical analysis (Fig. 2*A*) shows that in the 1980s, the lake was not P limited. Initial success at reducing the nutrient load did not have a strong impact on CHL until TP_lake_ fell below 40 µg⋅L^−1^, at which point CHL started responding to P limitation. In this way, the empirical relationship shown with the S-map is closely related to the increasing C:P ratio as a function of decreasing TP recently demonstrated in Swiss lakes undergoing reoligotrophication (37).

The effect of CHL on DO_B_, is quantified by the S-map coefficient estimating $\frac{\partial DO}{\partial \mathrm{CHL}}$ from the mechanistic embedding predicting DO_B_(*t* + 1) (*SI Appendix*, Fig. S6). During the eutrophic period, oxygen consumption was not sensitive to summer algal biomass (CHL) as $\frac{\partial DO}{\partial \mathrm{CHL}}∼0\;$ for TP_lake_ > 50 µg⋅L^−1^. With reoligotrophication and TP_lake_ < 50 µg⋅L^−1^, DO_B_ began to show a consistent negative response to changes in CHL (Fig. 2*B*). This complex, state-dependent response of DO_B_ to CHL is in agreement with the existing evidence that there has been an unfavorable food-web rearrangement under ongoing reoligotrophication (*SI Appendix*, Fig. S1) that has led to less organic matter transfer within the surface food web and high export to depth (30). In principle, such a trend could also be created by an increase in sediment oxygen uptake (which is not directly resolved in the empirical modeling); however, the current state of understanding the sediments of Lake Geneva does not suggest it (38). Either way, the state-dependent S-map coefficients measured on long-term time series provide an empirical quantification of the changes^†^ without having to explicitly resolve phytoplankton composition and food web.

## Management Insights

A long-term need for management is to understand the combined effects of climate change and reoligotrophication on the evolution of hypoxia over the coming decades. To first order, this can be accomplished by considering combined air temperature and TP scenarios.^‡^ In the traditional box-model framework, the oxygen dynamics reduce to combining a DO source term representing physical mixing with DO sink terms representing biogeochemical processes. The thermal structure relevant to oxygen dynamics in Lake Geneva is approximated by an equation-based deterministic model (Simstrat). This parameterized model has low error in reproducing the proximate physical dynamics of the lake from atmospheric forcing, able to predict daily thermocline depth across four decades with normalized root mean squared error (nrmse) of 6.7% (rmse = 21 m) (40). The S-map analysis shows a necessity for accounting for evolving nonlinear interactions in the biogeochemistry not represented in previous parameterized approaches (21, 41). Thus, we construct a hybrid approach, where the source term is equation-based and the sink term is represented by the empirically measured (equation-free) features captured by S-map regression (Fig. 2).

**Fig. 3.**
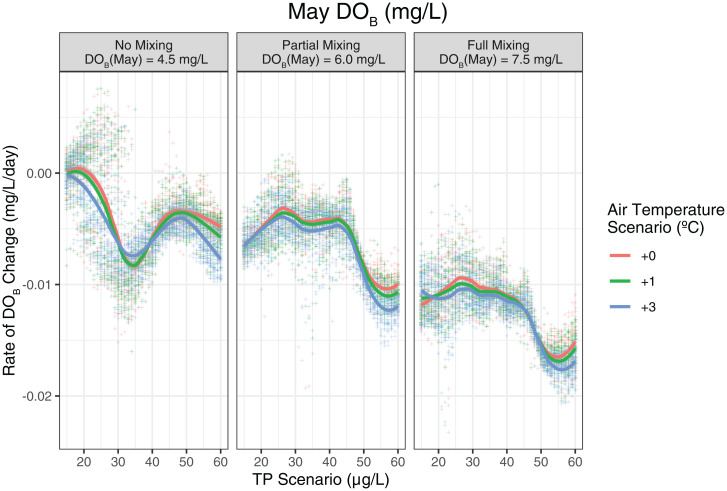
Short-term (6 mo) iterated EDM forecasts can be used to predict summer oxygen depletion under set temperature and phosphorus conditions. Here, the predictions are shown across three different postwinter DO_B_ concentrations (panel labels). Effectively, this figure is a graphical representation of the EDM calculations that are integrated in the hybrid-modeling pipeline (Fig. 4). In practice, summer stratified depletion forecasts can be linked to physical model predictions of winter mixing. Although the first-order mechanism of climate change affecting DO appears to be winter mixing, EDM shows that higher temperatures can also increase oxygen depletion in the summer, particularly when there has not been strong winter mixing (*Left*; May DO_B_ = 4.5 mg/L). The average deep-oxygen depletion for Lake Geneva from 1970 to 2012 has been previously estimated at ∼0.007 mg⋅L^−1^⋅d^−1^ (48) and falls in the middle of the range of the seasonally averaged (May through October) deep rate of oxygen depletion produced by EDM for these scenarios (0 to 0.017 mg⋅L^−1^⋅d^−1^). We only show results up to the year 2012, the last year with full deep mixing, in order to avoid bias in our data analysis between the currently low DO_B_ observed since 2013 and continuously decreasing TP. The results are very similar if data beyond 2012 are included to extrapolate behavior to even lower TP.

Conveniently, these processes separate seasonally. Thus, we focus on how the S-map relationships estimate the evolution of hypoxia over the 6-mo stratified periods following possible deep mixing in winter (Fig. 3). This plays to the strength of EDM, which typically performs best for short-term prediction of nonlinear systems (42) (*SI Appendix*, Fig. S4). Explicitly, we can consider alternative scenarios of management futures: different fixed-background TP concentrations (reoligotrophication states) and different predicted increases in air temperature (climate-change scenarios). With high DO_B_ at the onset of summer (DO_B_init_ = 7.5 and 6 mg⋅L^−1^), EDM experiments reveal a high DO_B_ depletion rate at high background TP (Fig. 3). The depletion rate gradually decreases with TP until TP ∼ 30 µg⋅L^−1^, but the empirical model suggests a reverse in the relationship at the lowest values of TP considered, meaning that further decreases in TP below ∼20 µg⋅L^−1^ lead to a reincreasing rate of DO_B_ depletion. In this way, the response of the lake to reoligotrophication appears not to follow (yet) the expected steady-state response between TP and DO_B_ shown in Müller et al. (32). Instead, the reversing trend at low TP is a product of the changing biogeochemical relationships shown in Fig. 2, which we interpret as a consequence of the previously documented unfavorable rearrangement of the food web—specifically, the emergence of less edible phytoplankton (13–15).

The simulated summer depletion in the absence of recent mixing (DO_B_init_ = 4.5 mg⋅L^−1^) shows the same features, with an additional reversal around 40 µg⋅L^−1^. This level of detail is hard to work out from a single lake time series, where the change in TP has happened in a long, slow, and steady way. It could instead be an artifact of limited observations in this stage of reoligotrophication due to the prolonged interval between mixing in the late 1980s and early 1990s (Fig. 1). However, it is consistent with the changing relationships quantified in Fig. 2—CHL is only weakly responding to phosphorous limitation (Fig. 2*A*), but causing increased oxygen demand at depth (Fig. 2*B*).

The above analysis shows that phosphorus levels dominate future behavior over air-temperature change, but only insofar as summer oxygen depletion is concerned. To capture the full scope for management, we combine the strengths of EDM with the equation-based physical model, Simstrat, to account for source and sink dynamics. The conceptual framework of the hybrid model is presented in *SI Appendix*, Fig. S3 and takes advantage of the way DO_B_ controls operate in distinct seasons. The meteorological forcing drives the physical Simstrat model that evolves an initial DO_B_ through the winter months; at the end of the mixing season, the data-driven EDM model is fed with deterministic model output (lake temperature and stratification) and TP_lake_ loading. Predictions of CHL and TP are made internal to the EDM component (using the S-map predictors in *SI Appendix*, Fig S3), avoiding the need of parameterizing the many possible relationships involved. These predictions are made iteratively for 6 mo, and then the DO_B_ at the end of October is fed back into Simstrat.

**Fig. 4.**
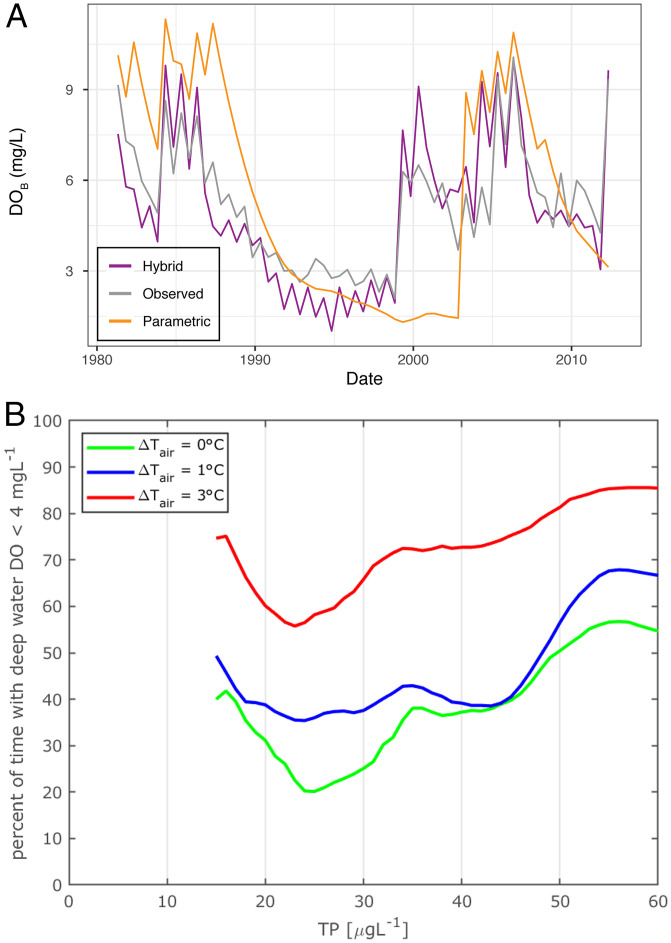
Hybrid-model predictions of DO_B_ forced by observed atmospheric and TP_lake_ and simulations under alternative scenarios. (*A*) The time series of hybrid-model-predicted DO_B_ under historical conditions (purple) is compared to observed DO_B_ (gray) and previous parametric efforts (orange) for reference. (*B*) Hybrid-model simulations are shown under alternative scenarios of atmospheric temperature and TP_lake_. The model is forced by the detrended historical time series, shifted by 0 °C (green), 1 °C (blue), and 3 °C (red) warming and a constant value of TP (TP_lake_). These simulations constitute a critical quantitative capability for management, linking physical model predictions of winter mixing and EDM predictions of summer DO depletion to examine the percentage of time that deep-water DO dips below 4 mg⋅L^−1^. Under 3 °C, the hybrid model estimates that the lake will be hypoxic the majority of the time, regardless of phosphorus levels. The less extreme (but increasingly unlikely) temperature scenarios suggest that the reoligotrophic lake state with the lowest TP loadings may not be optimal for lake health. Without broadening management actions (e.g., on the food web), further TP decreases are not predicted to benefit the lake.

The fundamental test for a model is if it can accurately predict (and not just fit) the observed dynamics of the system. The results in Fig. 4*A* show that the hybrid model using historical atmospheric forcing and a single initialization of the biogeochemistry is able to reproduce the temporal evolution of DO_B_ over 37 y, with high correlation between observed and predicted DO_B_ values (ρ = 0.89) and low error (mean absolute error = 0.94 mg⋅L^−1^). For comparison, equivalent predictions are also included using the traditional approach in Schwefel et al. (21) of fully parameterized physics and biogeochemistry. The substantially improved forecast skill of the hybrid model demonstrates an advantage to incorporating the emergent nonlinear effects between phosphorus, CHL, and oxygen that occur during reoligotrophication (Fig. 2).

The hybrid approach offers an improvement in bottom-line prediction and, more importantly, is capable of simultaneously accounting for changing air temperature and phosphorus loading as they act together through multiple, interdependent pathways. To diagnose and predict how DO will respond to the interplay of reoligotrophication and atmospheric warming over long time scales, we use the historical time-series observations (1981 through 2017) and translate them into hypothetical scenarios. This is accomplished by applying a simple offset in detrended air temperature and fixing TP to a certain value in the observed range. The hybrid model is then run for these 135 cases (i.e., 45 TP scenarios × 3 climate-change scenarios), producing a simulation of DO dynamics under each scenario. From these simulated dynamics, we calculate the percent of time with DO_B_ < 4 mg⋅L^−1^—i.e., a management benchmark for regulating deep water quality in Switzerland. Because all meteorological data besides air temperature are kept unchanged (*Materials and Methods*), this specific analysis does not aim to predict an exact future for the lake. However, other changes can be readily incorporated into scenario exploration and prediction as management relevance arises, including land-use changes or questions about other climatological effects.

Fig. 4 shows this management benchmark across all 135 scenarios and offers a number of insights on past and future management. First, the hybrid model demonstrates that the regulations related to TP reduction in Lake Geneva were effective at reducing the rate of oxygen depletion during the last 4 decades (decreasing trend in the percent of time with DO_B_ < 4 mg⋅L^−1^). The hybrid model predicts that under preintervention conditions (background TP ∼ 60 µg⋅L^−1^ and no change in median temperature, ΔT_air_ = 0 °C), the deepest 50 m of water would remain hypoxic 55% of the time. This value drops to 20% for a background TP ∼ 25 µg⋅L^−1^ (and ΔT_air_ = 0 °C). These estimates explain and buttress the initial success of the original single-factor management paradigm at addressing an “acute crisis,” where the extreme perturbation to TP so dominated the ecosystem in the early stages that other confounding considerations were relatively minor.

What does the hybrid model suggest about the more recent history and impending future, where lake complexity reemerges and the individual linear relationships among TP, CHL, and DO_B_ shift (Fig. 2)? Most importantly, the hybrid model suggests that the impact of moderate air-temperature increase (ΔT_air_ = 3 °C) on water quality would be on the same order as the eutrophication of the previous century. Thus, for ΔT_air_ = 3 °C and background TP ∼ 25 µg⋅L^−1^, the fraction of time with hypoxic deep water would be 55%, similar to the expectation for simulated high background TP (∼60 µg⋅L^−1^) with no warming (ΔT_air_ = 0 °C). Hypoxia would be even greater under 3 °C warming and unmitigated TP, with DO_B_ below the threshold 85% of the time. Moreover, the confounding effect of temperature may increase going forward, since according to the recent CH2018 scenario (Representative Concentration Pathway 8.5) for the western part of Switzerland, a temperature increase (ΔT_air_ = 3 °C) corresponds to the lower bound of predicted near-surface temperature increase for the period 2070 through 2100.

Finally, the hybrid model clarifies the limits that single-factor management through phosphorus mitigation alone has going forward. All three climate scenarios show counterintuitively that hypoxic conditions begin to increase again when TP < 25 µg⋅L^−1^ as a direct consequence of the state-dependent changes in the biogeochemical processes (Fig. 2). A decline in near-surface CHL from reoligotrophication can have unexpected effects on DO_B_ below TP < 25 µg⋅L^−1^ [e.g., as shifts in the food web or higher export fluxes (14, 30)]. Nevertheless, the conclusion is not that moderate pollution of the lake is acceptable; long sediment cores show that Lake Geneva was not hypoxic before the Anthropocene (31) with TP < 10 μg⋅L^−1^. Instead, this analysis demonstrates that, in addition to reducing nutrient loading, management will also need to identify other control levers to maintain a phytoplankton community and food web that limit oxygen depletion at depth.

The essential ingredients to the approach here were a parametric modeling framework that could account for physical processes controlling oxygen and multidecadal time series of biogeochemical variables that could be successfully modeled with EDM. Although Lake Geneva is one of the most well-studied lakes in the world, similar data exist for other deep lakes in the region, and many of these are already parameterized for Simstrat or other similar hydrodynamic models. A next step is to determine how reproducible the hybrid-model success is across a wider set of lake morphologies and ecological states. Moreover, combining observations from multiple lakes within a single EDM model of deep-lake biogeochemistry would enable informed extrapolation beyond the historical record of any one lake. However, the general principle of the hybrid approach should be applicable more broadly than just to deep lakes but also shallow lakes and even estuaries where physical processes are well represented in models and biogeochemical variables like CHL and nutrients have been measured through time.

Although this generalizability of approach is speculative, the need for practical inroads into modeling ecosystem complexity is not. While the initial correction from a single large ecosystem disturbance, be it excess nutrient loading or overfishing, can be managed based on common sense or a single conspicuous overriding relationship, the task becomes more complicated as we steer systems closer to their healthy states. Success will require finding practical avenues for addressing and even embracing complexity. We suggest that this, as well as contending with the ever-destabilizing effects of climate change, is the signature task for 21^st^-century environmental management.

## Materials and Methods

### Lake Geneva Time-Series Data and Reoligotrophication.

Lake Geneva (Lac Léman) is a deep (310 m), large (589 km^2^) lake located between Switzerland and France. Lake Geneva is arguably the cradle of European limnology due to the seminal interdisciplinary studies by Forel (24). It is classified as a warm monomictic lake with intermittent deep mixing. The last complete winter mixing was observed in 2012. All of the time-series data, except the Rhone River discharge, are from long-term monitoring conducted by the Centre Alpin de Recherche sur les Réseaux Trophiques des Ecosystèmes Limniques (CARRTEL) laboratory and freely available in the Système d’Observatoires, d’Expérimentations et de Recherche en Environnement Observatoire des Lacs (https://si-ola.inra.fr). TP_epi_ and CHL were volume-weighted averaged over the first 20 m of the water column. The total amount of phosphorus TP_lake_ was calculated by integrating over the hypsometry each bimonthly or monthly profile. Phosphorus concentration has been measured with two protocols, quantifying soluble-reactive phosphorus (SRP) and TP. However, these two measurements are tightly correlated (both integrated over the first 20 m or the full hypsometry) and give nearly identical results with EDM analysis. The SRP measurements give slightly higher predictive skill, however; thus, the results presented in the figures are those using SRP as an indicator of TP, rather than the measurement of TP. Rhone River data were obtained from the Swiss Federal Office for the Environment (https://www.hydrodaten.admin.ch/en/2009.html). Meteorological data were obtained from the Swiss Federal Office of Meteorology and Climatology, MeteoSwiss, for the monitoring station Pully (https://www.meteoswiss.admin.ch/home/measurement-values.html?param=messwerte-lufttemperatur-10min&station=PUY). The data derived from these sources needed for the calculations presented in the manuscript are included in *SI Appendix* and are also available at GitHub (https://github.com/SugiharaLab/Geneva_Hybrid) and Zenodo (43).

#### Climate change and reoligotrophication scenario.

This study did not use explicit regional climate predictions, such as those driven by greenhouse-gas-emission scenarios for changes in atmospheric forcing, or future land-use scenarios for predicting changes in nutrient loading. Instead, we reanalyzed the 30-y historical data by applying synthetic TP_lake_ and air-temperature time series. The air-temperature time series were generated by applying a uniform 0 °C, 1 °C, or 3 °C change to the detrended historical air temperature. The TP_lake_ time series were generated for a constant concentration from 65 to 15 µg⋅L^−1^ (1-µg⋅L^−1^ increments). The other meteorological drivers for Simstrat were unchanged, as we currently lack clear expectation for their fate under greenhouse-gas scenarios. The resulting Simstrat output for propagation of the air-temperature scenario into lake physics variables was then used to drive the EDM component. Our goal was to examine the benefits of a hybrid-modeling approach for addressing the complex challenges that will be confronting environmental management in the coming years. In principle, this same machinery can be generalized to follow any particular prediction of climate change or land-use.

### Equation-Based Model.

We used a 1D hydrodynamic model [Simstrat version (v)1.0 from https://github.com/Eawag-AppliedSystemAnalysis/Simstrat with an online near-real-time version at https://simstrat.eawag.ch/ (44)] to infer the evolution of the lake thermal structure based on meteorological (wind speed and direction, solar radiation, air temperature, relative humidity, and cloud coverage) and river (discharge and temperature) parameters. Simstrat combines a buoyancy-extended k-ε model with an internal seiche model. The model was recently improved to better reproduce deep mixing in deep lakes (41). The model was previously validated (21, 41) based on the in situ bimonthly to monthly profiles collected since 1957. Gaudard et al. (41) reported an rmse of ∼0.2 °C over 30 y over in the deep layer.

Resolving the dynamics of thermal structure of the lake allows for simple parametric relationships to approximate the mixing of surface water with dissolved oxygen at equilibrium with the atmosphere and deep water. Schwefel et al. (21) developed a two-box model of oxygen to run on output from Simstrat. The lake is divided into a surface layer and a deep layer, based on the time-varying thermocline depth (depth of maximum stratification indicated by the buoyancy frequency, N^2^). Additionally, there is a seasonally varying input of oxygen from the Rhone River, with river flux in summer entering the surface layer and in winter entering the deep layer.

### Equation-Free Model.

Comprehensive EDM analysis of DO and likely interactors was performed through a similar pipeline as analyses of planktonic food-web data in Deyle et al. (25) and by using rEDM 0.7.3 from GitHub (https://github.com/SugiharaLab) (a newer application programming interface is now available through the CRAN repository, https://cran.r-project.org/web/packages/rEDM/index.html). R-Markdown code to reproduce the EDM calculations is included in *SI Appendix* and is also available from GitHub (https://github.com/SugiharaLab/Geneva_Hybrid). First, univariate analysis with simplex (27) and S-map (29) confirmed the presence of low-dimensional, nonlinear dynamics in DO_B_ (*SI Appendix*). Thus, the basic assumption of EDM analysis is valid for these data.

Next, CCM was computed between all variables using simplex projection (26). Each pairwise test requires only a single fit parameter, the embedding dimension *E*. Following the insights of Ye et al. (45), we selected embedding dimension E* in [1,15] to maximize CCM skill at *tp* = 0 and then measure CCM skill at prediction-time *tp* = floor(−E*/2), the middle of the embedding vector. This mediates the risk of statistical overfitting without relying on univariate estimates of *E* that can be too low.

Multivariate EDM analysis (37, 46) was used to examine forecast improvement when potential drivers are added to the embedding (set of coordinate variables). This provides an additional test of causality overlapping, but not identical to, CCM (28). When a potential driver *X_j_* improves prediction of target *X_i_*, it can indicate that *X_j_* interacts with *X_i_*. When multiple potenital drivers are added in sequence, comparing improvments in prediction skill can additionally tease out when variables act interdependently from when one driver acts indirectly through another (28). Thus, multivariate S-map prediction was used to examine whether physical and biogeochemical drivers of DO act interdependently. S-map analysis on multivariate empirical models can also be used to characterize changing interactions (25). S-map models for predicting CHL, TP_epi_, and DO_B_ were built from step-by-step multivariate EDM analyses (*SI Appendix*, Figs. S3 and S4), where the ultimate predictor variables were chosen to maximize forecast skill.

Finally, these multivariate models for predicting DO_B_ were used for scenario exploration of climate change and management futures. While EDM scenario exploration has normally involved single time-step predictions (47), the analysis here uses iterated short-term prediction to generate long-term behavior on a time scale relevant to management.

### Hybrid Model.

We provide here an initial attempt to couple equation-based and equation-free models (*SI Appendix*, Fig. S4). The rationale is to use an equation-based model for physical processes and an equation-free model for biogeochemical processes. The thermal structure is provided by the equation-based model to the equation-free model. Both physical and biogeochemical results are merged together into a simple box model. The temporal evolution of the box size is given by the thermocline depth (equation-based model). The temporal evolution of DO in the upper box was estimated following equation 7 in Schwefel et al. (21), except that we used the CHL output from EDM instead of synthetic averaged CHL values. From initial conditions of DO at the surface and at the bottom in May 1981, we used the results of the EDM (given the temperature scenario, the reoligotrophication scenario, deep mixing depth, and initial DO_B_) to predict the rate of oxygen depletion in the deep water over 180 d. During the following winter, reoxygenation was estimated based on the extent of the deep winter mixing and the relative concentration of oxygen in the upper and lower layer, as well as the Rhone River underflow (40) providing oxygenated water to the deep layer.

## Supplementary Material

Supplementary File

Supplementary File

## Data Availability

R scripts (*.R), R markdown (*.rmd), R data files (*.Rdata), text data files (*.csv; *.dat), and text parameter files (*.par) have been deposited in GitHub (https://github.com/SugiharaLab/Geneva_Hybrid; https://github.com/SugiharaLab) and Zenodo (43).

## Footnotes

*The limnological consequences of other aspects of climate change, such as changing precipitation patterns across the lake watershed, are difficult to generalize or even predict, particularly as they can depend on future management and land use.

^†^Whether or not this rearrangement of the food web is transient or permanent.

^‡^Recent downscaled climate scenarios for Switzerland affirm that air temperature is the single climate parameter in the area that will experience unambiguous increase or decrease under greenhouse-gas scenarios of the next century (39). Though systematic increases or decreases in, e.g., rainfall or wind speed would likely impact DO, there are no coherent scenario predictions of change in these variables to explore at this point.
