# Supplementary material for: A hybrid empirical and parametric approach for managing ecosystem complexity: Water quality in Lake Geneva under nonstationary futures
Source: Proc Natl Acad Sci U S A. 2022 Jun 22;119(26):e2102466119. doi: 10.1073/pnas.2102466119 (PMC9245694; doi:10.1073/pnas.2102466119)
Supplement: Supplementary File [file pnas.2102466119.sapp.pdf]

PNAS
www.pnas.org

Supplementary Information for

A hybrid empirical and parametric approach for managing ecosystem complexity: water quality in Lake Geneva under nonstationary futures.

Ethan Deyle[a,b,1,2], Damien Bouffard[c,1,2], Victor Frossard[d], Robert Schwefel[e,f], John Melack[e,g], George Sugihara[b,1,2]

[a] Department of Biology, Boston University, Boston, MA 02215

[b] Scripps Institution of Oceanography, University of California San Diego, La Jolla, CA 92093

[c] Department of Surface Waters – Research and Management, Eawag - Swiss Federal Institute of Aquatic Science and Technology, Kastanienbaum, 6047, Switzerland

[d] Centre Alpin de Recherche sur les Réseaux Trophiques des Ecosystèmes Limniques, Univ. Savoie Mont Blanc, INRAE, Thonon-les-Bains, 73376, France

[e] Department of Ecology, Evolution, and Marine Biology, University of California Santa Barbara, Santa Barbara, CA 93106

[f] Department of Ecohydrology and Biogeochemistry, Leibniz-Institute of Freshwater Ecology and Inland Fisheries, Berlin, 12587, Germany;

[g] Bren School of Environmental Science & Management, University of California Santa Barbara, Santa Barbara, CA 93106

[1] Contributed equally as primary authors

[2] Corresponding authors Ethan Deyle, Damien Bouffard, George Sugihara

Email: *edeyle@bu.edu*, *Damien.Bouffard@eawag.ch*, *gsugihara@ucs.edu*

This PDF file includes:

Supplementary text
Tables S1
Figures S1 to S6
SI References

Other supplementary materials for this manuscript include the following:

Datasets S1

Supplementary Information Text

Additional detail on the history of Lake Geneva.

The history of Lake Geneva is an iconic example where eutrophication from runoff gave way to partial recovery through control of anthropogenic phosphorus inputs, but where the extent of recovery has not met expectation. Although lake-averaged TP has been reduced by a factor of 6 from up to ~90 μg L^{-1} in 1980 to ~15 μg L^{-1} today (Figure 1B), this TP reduction did not result in decrease in lake averaged CHL or change in oxygen depletion rate (1).

The complex response to re-oligotrophication is tentatively explained by an interdependent intersection of chemical, biological and physical processes, some well documented, some more hypothetical. These are graphically summarized in Figure S1. First, TP internal loading from the sediments complicates any attempt to simply correlate TP external loading and lake averaged TP (2, 3) despite earlier seminal work suggesting a simple first-order relationship (4). Second, ecological observations during the re-oligotrophication period suggest significant changes in phytoplankton composition and productivity (5, 6), zooplankton community structure (7) and the fish community (8) – food-web alterations thought to result from complex, coupled effects of top-down and bottom-up controls (9). Third, increasing air temperature (the most clear effect of climate change for the Lake Geneva area) contributes to modify the thermal structure and study (10) suggests also promotes less edible harmful cyanobacteria. The change in thermal structure also leads to prolonged duration of the summer stratified period and decrease in the frequency of deep mixing event (11, 1, 12).

R-Markdown Code

Below are the key steps of data processing and computation for reproducing EDM and hybrid-model calculations in the main text. The full materials for calculations are available at the GitHub URL listed in the text, https://github.com/SugiharaLab/Geneva_Hybrid. In several cases

we have condensed the code by calling functions to perform auxiliary tasks like data curation and complex plotting. These are contained in the helper scripts sourced below, but need to be retrieved from the GitHub by readers who desire to reproduce the full plots.

Also, please note we have made liberal use of the pipe operator “%>%” from the magrittr package. This operator takes the left-hand side as the first argument to right hand side, so “x %>% f” is equivalent to “f(x)” and “x %>% f(y,z,…)” is equivalent to “f(x,y,z,…)”.

This project was completed using Version 0.7.3 of rEDM. Since there were substantial changes to syntax with the advent of rEDM 1.0, it is easiest to simply install the old version in a local "lib" folder for this project. This will require most machines to have Rtools installed in the default location.

```
library(' remotes')

install_version("rEDM", version = "0.7.3",lib = "./lib")
```

The first step is to load the required packages, including the archived 0.7.3 version of rEDM.

```
library('tidyverse')

library('lubridate')

library("rEDM",lib.loc = "./lib")

sessionInfo()

## R version 3.6.0 (2019-04-26)
## Platform: x86_64-apple-darwin15.6.0 (64-bit)
## Running under: macOS Mojave 10.14.6
##
## Matrix products: default
## BLAS:   /Library/Frameworks/R.framework/Versions/3.6/Resources/lib/libRbla
s.0.dylib
## LAPACK: /Library/Frameworks/R.framework/Versions/3.6/Resources/lib/libRlap
ack.dylib
##
## locale:
## [1] en_US.UTF-8/en_US.UTF-8/en_US.UTF-8/C/en_US.UTF-8/en_US.UTF-8
##
## attached base packages:
## [1] stats     graphics  grDevices utils     datasets  methods   base
```

```
##
## other attached packages:
##  [1] rEDM_0.7.3      lubridate_1.7.4 forcats_0.4.0   stringr_1.4.0
##  [5] dplyr_1.0.7     purrr_0.3.3     readr_1.3.1     tidyr_1.1.3
##  [9] tibble_3.1.4    ggplot2_3.3.0   tidyverse_1.3.0
```

We additionally source a set of help functions to neaten plot creation.

```
source('./FUNCTIONS/F_data_and_plots.R')
```

Preamble

The EDM calculations in the manuscript include as explanatory variables two outputs of the parametric physical model, Simstrat. Thus, it is necessary to first run Simstrat. Since the ease of installation will be platform dependent, we include the direct output of thermal structure as a supplemental file. The function “Simstrat_physics_initial()” is used, and is capable of generating the output file on a Windows machine that can run the archived binary of Simstrat. The function then calculates midnight values of the height of the mixed layer, h_mix, and surface temperature, T_surf.

```
data_file <- "./INPUTS/EDM_input_data.Rdata"

if(!file.exists(data_file)){
  load("./DATA/lake_geneva_observ.Rdata")

t_interp <- lake_geneva_interp$date

Simstrat_out <- Simstrat_physics_inital(run=FALSE,
                                        file.output = './Outputs/LakeGeneva/T_out.dat
')

Simstrat_out_reduced <- Simstrat_out %>%
  rename(date = time) %>%
  mutate(date = ymd(date)) %>%
  mutate(date = cut(date,breaks=c(min(t_interp)-days(30),t_interp),right=TRUE,labels=
lag(t_interp)) %>% as.Date() ) %>%
  group_by(date) %>%
  # can calculate h_mix at midnight
  summarise(h_mix_model = max(h_mix,na.rm=T), T_surf_model = mean(T_surf,na.rm=T))

data_lake_geneva <- full_join(
  lake_geneva_interp,
  Simstrat_out_reduced,
  by = "date"
)
```

```r
save(data_lake_geneva,file=data_file)
}

load(data_file)
```

This yields the following variables for our primary analysis:

```r
names(data_lake_geneva)

##  [1] "date"         "oxydeep"      "T_surf"       "T_bot"
##  [5] "T_delta"      "h_mix"        "Ptot_lake"    "Ptot_epi"
##  [9] "PO4_lake"     "PO4_epi"      "chl"          "wind_speed"
## [13] "T_air"        "rhone"        "T_surf_model" "h_mix_model"
```

Finally, the hybrid model contains an EDM predictor for DO over six-month intervals. To do this, we need to expand on the basic tools of the rEDM package and define a function that performs iterative multivariate EDM forecasts of DO. We define equivalent functions for both simplex and S-map EDM predictors that have the same general structure. The S-map predictor is used for historical analysis, but due to its higher computational overhead, iterative forecasting for the hybrid model was done with the less computationally intensive simplex predictor.

```r
sim_EDM_smap_diff <- function(block_train,
                              block_sim,
                              t_sim,
                              tp = 1,
                              theta = 0,
                              num_neighbors="e+1",
                              lib_train = c(1,NROW(block_train)),
                              pred_sim = c(1,NROW(block_sim)),
                              sim_col = NULL, # could set to which columns are NAs
                              predictor_col = 1:( NCOL(block_train) - 1 ),
                              ...){

  n_var <- NCOL(block_train) - 1

  # Normalize and take differences
  v_norms <- block_train %>%
    summarise_all(list(~sd(.,na.rm=T)))

  block.t_norm <- bind_rows( block_train %>%
                               mutate_at( set_names(sim_col,sim_col), list(delta = ~
c(NA,diff(.)))  ) %>%
                               mutate(!!!imap(v_norms[-1],function(col_norm, col_name
, data) data[[col_name]]/col_norm,.)) ,
                             block_sim %>%
                               mutate_at( set_names(sim_col,sim_col), list(delta = ~
c(NA,diff(.)))  ) %>%
                               mutate(!!!imap(v_norms[-1],function(col_norm, col_name
, data) data[[col_name]]/col_norm,.))
```

```
  )

  # Fit S-map thetas to prediction of first-differenced values
    if(length(theta)>1){
        theta.star <- rep(NA,n_var)
        for(i.col in sim_col){

            out.temp <- do.call(bind_rows,lapply(theta,function(theta.i){
                block_lnlp(block.t_norm,lib=lib_train,pred=lib_train,
                           method = 's-map',
                           theta = theta.i,num_neighbors = 0,
                           columns=predictor_col,
                           target_column = paste0(i.col,"_delta"),
                           first_column_time = TRUE,
                           ...)
            }))

            theta.star[i.col] <- theta[which.max(out.temp$rho)]
        }}else{
            theta.star=rep(theta,n_var)
        }

  # Iterate through time
  t_pred <- 1
  while(t_pred < t_sim + 1){
    t_pred <- t_pred+1
    delY <- as.data.frame(array(dim=c(1,n_var),dimnames=list(NULL,names(block_train)[
-1])))

    lib_temp <- lib_train

    # Evaluate simplex projection
    for(i.col in sim_col){

      out.temp <- block_lnlp(block.t_norm,
                             method='s-map',
                             lib=lib_temp,
                             pred=c((NROW(block_train))+(t_pred-1),(NROW(block_train)
)+t_pred),
                             columns=predictor_col,
                             theta=theta.star[i.col],
                             target_column = paste0(i.col,"_delta"),
                             num_neighbors = 0,
                             stats_only = FALSE,
                             silent = TRUE,
                             first_column_time = TRUE,
                             ...)

      delY[i.col] <- out.temp$model_output[[1]]$pred[1]

      # REPLACE IN BLOCK BY ADDING TO x_i.col(t_pred-1)
      block.t_norm[NROW(block_train)+t_pred,paste0(i.col,"_delta")] <- delY[i.col]
      block.t_norm[NROW(block_train)+t_pred,i.col] <- block.t_norm[NROW(block_train)+
t_pred-1,i.col] + delY[i.col]
```

```
    } # for(i.col)
  }

  # Undo normalize
  block_sim_out <- block.t_norm[(NROW(block_train))+(1:t_sim),] %>%
    mutate(!!!imap(v_norms[-1],function(col_norm, col_name, data) data[[col_name]]*co
l_norm,.))

  return(
    bind_rows( block_sim_out %>% mutate(type = 'sim'), #time=lib[2]+ 1:t_sim,
               block_sim[1+(1:t_sim),] %>% mutate(type = 'true') ) #time=lib[2]+ 1:t_
sim,
  )
} # sim_EDM_smap_diff

sim_EDM_simplex_diff <- function(block_train,
                                 block_sim,
                                 t_sim,
                                 tp = 1,
                                 num_neighbors="e+1",
                                 lib_train = c(1,NROW(block_train)),
                                 pred_sim = c(1,NROW(block_sim)),
                                 sim_col = NULL, # could set to which columns are NAs
                                 predictor_col = 1:( NCOL(block_train) - 1),
                                 ...){

  n_var <- NCOL(block_train) - 1

  v_norms <- block_train %>%
    summarise_all(list(~sd(.,na.rm=T)))

  block.t_norm <- bind_rows( block_train %>%
                               mutate_at( set_names(sim_col,sim_col), list(delta = ~
c(NA,diff(.)))  ) %>%
                               mutate(!!!imap(v_norms[-1],function(col_norm, col_name
, data) data[[col_name]]/col_norm,.)) ,
                             block_sim %>%
                               mutate_at( set_names(sim_col,sim_col), list(delta = ~
c(NA,diff(.)))  ) %>%
                               mutate(!!!imap(v_norms[-1],function(col_norm, col_name
, data) data[[col_name]]/col_norm,.))
  )

  t_pred <- 1
  while(t_pred < t_sim + 1){
    t_pred <- t_pred+1
    delY <- as.data.frame(array(dim=c(1,n_var),dimnames=list(NULL,names(block_train)[
-1])))

    lib_temp <- lib_train

    for(i.col in sim_col){

      out.temp <- block_lnlp(block.t_norm,
                             method='simplex',
```

```
                                lib=lib_temp,
                                pred=c((NROW(block_train))+(t_pred-1),(NROW(block_train)
)+t_pred),

                                columns=predictor_col,
                                target_column = paste0(i.col,"_delta"),
                                num_neighbors = num_neighbors,
                                stats_only = FALSE,
                                silent = TRUE,
                                first_column_time = TRUE,
                                ...)

      delY[i.col] <- out.temp$model_output[[1]]$pred[1]

      block.t_norm[NROW(block_train)+t_pred,paste0(i.col,"_delta")] <- delY[i.col]
      block.t_norm[NROW(block_train)+t_pred,i.col] <- block.t_norm[NROW(block_train)+
t_pred-1,i.col] + delY[i.col]

    } # for(i.col)
  }
    block_sim_out <- block.t_norm[(NROW(block_train))+(1:t_sim),] %>%
    mutate(!!!imap(v_norms[-1],function(col_norm, col_name, data) data[[col_name]]*co
l_norm,.))

  return(
    bind_rows( block_sim_out %>% mutate(type = 'sim'), #time=lib[2]+ 1:t_sim,
               block_sim[1+(1:t_sim),] %>% mutate(type = 'true') ) #time=lib[2]+ 1:t_
sim,
  )
} # sim_EDM_simplex_diff
```

We define a third function that performs predictions on values of the target, rather than first differences. This is used for simulating *chl* and *PO4_epi*.

```
sim_EDM_smap <- function(block_train,
                         block_sim,
                         t_sim,
                         tp = 1,
                         lib_train = c(1,NROW(block_train)),
                         pred_sim = c(1,NROW(block_sim)),
                         sim_col = NULL, # could set to which columns are NAs
                         theta = 1,
                         predictor_col = 1:( NCOL(block_train) - 1 ),
                         ...){

  n_var <- NCOL(block_train) - 1

  block.t <- bind_rows(block_train,
                       block_sim)

  v_norms <- block_train %>%
    summarise_all(list(~sd(.,na.rm=T)))

  block.t_norm <- bind_rows( block_train %>%
                              # mutate_at( set_names(sim_col,sim_col), list(delta =
```

```r
~ c(NA,diff(.)))  ) %>%
                                mutate(!!!imap(v_norms[-1],function(col_norm, col_name
, data) data[[col_name]]/col_norm,.)) ,
                              block_sim %>%
                                # mutate_at( set_names(sim_col,sim_col), list(delta =
~ c(NA,diff(.)))  ) %>%
                                mutate(!!!imap(v_norms[-1],function(col_norm, col_name
, data) data[[col_name]]/col_norm,.))
  )

  #### fit S-map thetas to prediction of each simulation column
  if(length(theta)>1){
    theta.star <- rep(NA,n_var)
    for(i.col in sim_col){

      out.temp <- do.call(bind_rows,lapply(theta,function(theta.i){
        block_lnlp(block.t_norm,lib=lib_train,pred=lib_train,
                   method = 's-map',
                   theta = theta.i,num_neighbors = 0,
                   columns=predictor_col,
                   target_column = i.col,
                   first_column_time = TRUE,
                   ...)
      }))

      theta.star[i.col] <- theta[which.max(out.temp$rho)]
    }}else{
      theta.star=rep(theta,n_var)
    }


  t_pred <- 1

  while(t_pred < t_sim + 1){
    t_pred <- t_pred+1
    Y <- as.data.frame(array(dim=c(1,n_var),dimnames=list(NULL,names(block_train)[-1]
)))

    lib_temp <- lib_train

    for(i.col in sim_col){

      out.temp <- block_lnlp(block.t_norm,
                             method='s-map',
                             lib=lib_temp,
                             pred=c((NROW(block_train))+(t_pred-1),(NROW(block_train)
)+t_pred),
                             columns=predictor_col,
                             target_column = i.col,
                             theta=theta.star[i.col],
                             num_neighbors = 0,
                             stats_only = FALSE,
                             silent = TRUE,
                             first_column_time = TRUE,
                             ...)
```

```
      Y[i.col] <- out.temp$model_output[[1]]$pred[1]

      block.t_norm[NROW(block_train)+t_pred,i.col] <- Y[i.col]
    } # for(i.col)
  }

  block_sim_out <- block.t_norm[(NROW(block_train))+1+(1:t_sim),] %>%
    mutate(!!!imap(v_norms[-1],function(col_norm, col_name, data) data[[col_name]]*co
l_norm,.))

  return(
    bind_rows( block_sim_out %>% mutate(type = 'sim'), #time=lib[2]+ 1:t_sim,
               block_sim[1:t_sim,] %>% mutate(type = 'true') ) #time=lib[2]+ 1:t_sim,
  )
} # sim_EDM_smap
```

CCM calculations

We generate CCM measurements to identify coupling using the purrr function *map_df*() to loop through candidates and arrange the results in a single data-frame. Note that the direction of CCM is counter to the direction of the indicated coupling, i.e. response variables will cross-map driver variables.

```
ccm_block <- data_lake_geneva %>%
  select(-date)

out.CCM_DO_delta <- map_df(names(ccm_block ),function(j){

    data <- ccm_block %>%
        mutate(DO_delta = c(NA,diff(oxydeep)))

    ccm.temp <- do.call(bind_rows, lapply(1:10,function(E) {
        ccm(data,lib_column = 'DO_delta',target_column = j,
            E = E, tp = -1,
            num_samples = 1,lib_sizes=NROW(data),random_libs = F)
        }))

    Estar = ccm.temp$E[which.max(ccm.temp$rho)[1]]

    ccm(data,lib_column = 'DO_delta',target_column = j,
        E = Estar, tp = -floor(Estar/2),
        num_samples = 1,lib_sizes=NROW(data),random_libs = F)
})
```

These outputs are then used to Table S1.

```
t_S1 <- out.CCM_DO_delta %>%
    filter(target_column!="oxydeep") %>%
    select(target_column,num_pred,rho) %>%
    mutate(rho=signif(rho,digits=3)) %>%
```

```
    arrange(-rho) %>%
    rename(Driver=target_column,`Cross-map skill ()`=rho)

print(t_S1)
```

```
##          Driver num_pred Cross-map skill ()
## 1   h_mix_model      524              0.574
## 2         T_air      524              0.553
## 3       T_delta      526              0.522
## 4        T_surf      526              0.512
## 5  T_surf_model      524              0.498
## 6         rhone      524              0.403
## 7         h_mix      528              0.375
## 8         T_bot      526              0.353
## 9       PO4_epi      524              0.308
## 10     Ptot_epi      524              0.301
## 11   wind_speed      524              0.201
## 12     PO4_lake      524              0.187
## 13    Ptot_lake      524              0.176
## 14          chl      478              0.135
```

We also calculate cross-map skill for the dynamic biogeochemistry variables, *chl* and *TP_surf*.

These results are the basis for the table panels of Figure S4.

```
ccm_block <- data_lake_geneva %>%
    mutate(year_sine=sin(2*pi*yday(date)/365)) %>%
    mutate(T_diff_model=T_air-T_surf_model) %>%
  select(-date)

out.CCM_chl <- map_df(names(ccm_block ),function(j){

    data <- ccm_block

    ccm.temp <- do.call(bind_rows, lapply(1:10,function(E) {
        ccm(data,lib_column = 'chl',target_column = j,
            E = E, tp = -1,
            num_samples = 1,lib_sizes=NROW(data),random_libs = F)
        }))

    Estar = ccm.temp$E[which.max(ccm.temp$rho)[1]]

    ccm(data,lib_column = 'chl',target_column = j,
        E = Estar, tp = -floor(Estar/2),
        num_samples = 1,lib_sizes=NROW(data),random_libs = F)
})

out.CCM_po4 <- map_df(names(ccm_block ),function(j){

    data <- ccm_block

    ccm.temp <- do.call(bind_rows, lapply(1:10,function(E) {
        ccm(data,lib_column = 'PO4_epi',target_column = j,
            E = E, tp = -1,
            num_samples = 1,lib_sizes=NROW(data),random_libs = F)
```

```
        }))

    Estar = ccm.temp$E[which.max(ccm.temp$rho)[1]]

    ccm(data,lib_column = 'PO4_epi',target_column = j,
        E = Estar, tp = -floor(Estar/2),
        num_samples = 1,lib_sizes=NROW(data),random_libs = F)

})

t_S5A <- out.CCM_chl %>%
    filter(target_column!="chl") %>%
    select(target_column,num_pred,rho) %>%
    mutate(rho=signif(rho,digits=3)) %>%
    arrange(-rho) %>%
    rename(Driver=target_column,`Cross-map skill ()`=rho)

print(t_S5A)

##          Driver num_pred Cross-map skill ()
## 1     year_sine      485             0.8230
## 2  T_surf_model      485             0.8060
## 3         T_air      485             0.7820
## 4        T_surf      485             0.7760
## 5       T_delta      485             0.7760
## 6  T_diff_model      485             0.6790
## 7         rhone      485             0.6600
## 8   h_mix_model      491             0.6230
## 9       PO4_epi      485             0.6100
## 10     Ptot_epi      485             0.5900
## 11        h_mix      491             0.5720
## 12     PO4_lake      485             0.5370
## 13    Ptot_lake      485             0.5310
## 14   wind_speed      487             0.2060
## 15        T_bot      485             0.1180
## 16      oxydeep      491             0.0225

t_S5B <- out.CCM_po4 %>%
    filter(target_column!="PO4_epi") %>%
    select(target_column,num_pred,rho) %>%
    mutate(rho=signif(rho,digits=3)) %>%
    arrange(-rho) %>%
    rename(Driver=target_column,`Cross-map skill ()`=rho)

print(t_S5B)

##          Driver num_pred Cross-map skill ()
## 1      Ptot_epi      542              0.975
## 2     Ptot_lake      536              0.968
## 3      PO4_lake      536              0.967
## 4     year_sine      536              0.961
## 5  T_surf_model      536              0.946
## 6         T_air      536              0.944
## 7        T_surf      536              0.940
## 8       T_delta      536              0.938
```

```
## 9   h_mix_model      537              0.864
## 10 T_diff_model      536              0.853
## 11        rhone      536              0.814
## 12        h_mix      538              0.769
## 13        T_bot      536              0.617
## 14          chl      489              0.522
## 15   wind_speed      536              0.487
## 16      oxydeep      535              0.318
```

Short-term Multivariate forecasting

To perform short-term multivariate EDM forecasting analysis, we define functions to make calls to the rEDM function block_lnlp() to perform the short-term multivariate forecasting analyses across lists of specificed embedding coordinates.

```
do_mEDM_models <- function(block,L_models,target_var='oxydeep',tp=0,exclusion_radius=
6){

  theta_list <- c(0, 1e-04, 3e-04, 0.001, 0.003, 0.01, 0.03, 0.1, 0.3, 0.5, 0.75, 1,
1.5, 2, 3, 4, 6, 8)

  results_mEDM <- map(L_models,function(L){

    multi_stats <- map_df(theta_list, function(theta.i)
      block_lnlp(block = block,
                 target_column = match(target_var,names(block)),
                 columns = match(L,names(block)),
                 tp = tp,
                 lib = c(1,NROW(block)),
                 pred = c(1,NROW(block)),
                 method = 's-map',
                 theta = theta.i,
                 num_neighbors = 0,
                 exclusion_radius = exclusion_radius,
                 stats_only = TRUE) )

    theta.i <- multi_stats$theta[which.max(multi_stats$rho)]

    multi_preds <- block_lnlp(block = block,
                              target_column = match(target_var,names(block)),
                              columns = match(L,names(block)),
                              tp = tp,
                              method = 's-map',
                              theta = theta.i,
                              num_neighbors = 0,
                              exclusion_radius = exclusion_radius,
                              stats_only = FALSE)$model_output[[1]]



    return(list(multi_stats=multi_stats,
                multi_preds=multi_preds))
```

```
  } # function(L)
  ) # map(L_models)

  results_mEDM <- transpose(results_mEDM)

} #do_mEDM_models
```

Using this function, we create an additional function to performer sequential mEDM analysis with “greedy” variable selection.

```
do_mEDM_greedy <- function(block,embed0,L_variables,target_col=1,tp=1,max_E=length(L_
variables),...){

  result_embed0 <- do_mEDM_models(block=block, list(embed0),target_var=target_col,tp=
tp,...) %>%
    {do.call(bind_rows,.$multi_stats)} %>%
    mutate(embedding_label=label_embeddings(embedding,
                                            names(block),
                                            dictionary = dictionary_extended) %>% as.
factor()) %>%
    mutate(embedding=embedding_int_to_chr(embedding,names(block)))

  g_steps <- vector(mode = 'list')

  while(T){

    L_models_greedy <- map(setdiff(L_variables,embed0),~c(embed0,.))

    df_RESULT_i <- do_mEDM_models(block=block, L_models_greedy,target_var=target_col,
tp=tp,...) %>%
      { do.call(bind_rows,.$multi_stats)} %>%
      mutate(embedding_label=label_embeddings(embedding,
                                              names(block),
                                              dictionary = dictionary_extended) %>% a
s.factor()) %>%
      mutate(embedding=embedding_int_to_chr(embedding,names(block)))

    g_step_i <- df_RESULT_i %>%
      ggplot(aes(x=theta,y=rho,color=embedding_label)) +
      geom_line(lwd=.75) +
      geom_line(data=result_embed0,lwd=1) +
      theme_bw()
    g_steps <- c(g_steps,g_step_i)

    if(max(result_embed0$rho) > max(df_RESULT_i$rho)){
      break
    } # if (max rho)

    embed0 <- df_RESULT_i %>% top_n(1,rho) %>% pull(embedding) %>% unlist
    result_embed0 <- df_RESULT_i %>% filter(paste(embedding)==paste(list(embed0)))

    if(length(embed0) >= max_E){
      break
    } # if length(embed0)
```

```
  } #  while(T)

  return(result_embed0)
} # function(do_mEDM_greedy)
```

Finally, note it is important to normalize inputs before multivariate analyses so that distances in each observational variable are roughly equivalent. Thus we define a second data frame with normalized observations.

```
data_lake_geneva_norm <- data_lake_geneva %>%
  select(-date) %>%
  mutate(oxydeep_delta = c(NA,diff(oxydeep))) %>%
  mutate_at(vars(-starts_with('oxy')),funs(./sd(.,na.rm=TRUE)))

## Warning: `funs()` was deprecated in dplyr 0.8.0.
## Please use a list of either functions or lambdas:
##
##   # Simple named list:
##   list(mean = mean, median = median)
##
##   # Auto named with `tibble::lst()`:
##   tibble::lst(mean, median)
##
##   # Using lambdas
##   list(~ mean(., trim = .2), ~ median(., na.rm = TRUE))
```

The initial multivariate EDM experiment for DO was designed to test the importance of biogeochemical drivers in predicting behavior, and the outputs are used to create Figure 1D. Note in this first analysis, DO in the previous time-step is not included as a possible predictor.

```
target_var <- 'oxydeep'

L_models <- list( c('h_mix','T_surf','T_air','rhone'),
                  c('h_mix','T_surf','T_air','rhone','chl'),
                  c('h_mix','T_surf','T_air','rhone','chl','PO4_epi'),
                  c('h_mix','T_surf','T_air','rhone','chl','PO4_lake'),
                  c('h_mix','T_surf','T_air','rhone','chl','PO4_epi','PO4_lake') )

results_mEDM_exp1 <- do_mEDM_models(block=data_lake_geneva_norm,L_models=L_models,tar
get_var=target_var,exclusion_radius=0)

RESULTS_figure_1D <- do.call(bind_rows,results_mEDM_exp1$multi_stats) %>%
    mutate(embedding=label_embeddings_parsable(embedding,
                                       names(data_lake_geneva_norm),
                                       dictionary = dictionary_expressions) %>% as.fac
tor())

f_q_exp <- function(breaks) { parse(text=breaks)}

label_list <- map(unique(RESULTS_figure_1D$embedding),~ bquote(.))
```

```
RESULTS_figure_1D %>%
    mutate(embedding = factor(embedding,
                              levels = unique(embedding)[rank(str_count( unique(embed
ding),","),ties.method = 'first')])) %>%
    ggplot(aes(x=theta,y=rho,color=embedding)) + geom_line(lwd=1) +
    scale_color_viridis_d(labels= f_q_exp) +
    labs(x='Nonlinearity (\u03B8)',y='Forecast Skill (\u03C1)',color=NULL) + theme_bw
() + theme(legend.position = "bottom",legend.text=element_text(size=7,hjust=0)) + gui
des(color=guide_legend(nrow=5,byrow=TRUE))
```

Multivariate EDM analysis of chlorophyll was used to characterize changing biogeochemical interactions, and provide simulations of chlorophyll under forcing scenarios for the hybrid model analysis.

```
block_chl_mEDM <- data_lake_geneva %>%
    mutate(year_sine=sin(2*pi*yday(date)/365)) %>%
    mutate(year_cosine=cos(2*pi*yday(date)/365)) %>%
    filter(date>="1976-05-08") %>%
    mutate_at(vars(-date),list(~./sd(.,na.rm=TRUE)))

data_univar <- data_lake_geneva %>% filter(date>="1976-05-08") %>% pull(chl)

fname.chl_greedy <- './OUTPUTS/mEDM_chl_greedy.Rdata'

if(!file.exists(fname.chl_greedy)){
  L_candidate_variables <- c('year_sine','chl','rhone','h_mix_model','PO4_epi')

  results_mEDM_greedy <- do_mEDM_greedy(block_chl_mEDM,embed0=c('T_surf_model','PO4_l
ake'),L_candidate_variables,target_col="chl",max_E=5)
  save(results_mEDM_greedy,file=fname.chl_greedy)
}else{
  load('./OUTPUTS/mEDM_chl_greedy.Rdata')
}

results_mEDM_greedy %>% select(embedding,theta,rho,mae,rmse) %>% print()

##                            embedding  theta       rho       mae      rmse
## 1  T_surf_model, PO4_lake, year_sine 0.0000 0.3997000 0.6516280 0.9167866
## 2  T_surf_model, PO4_lake, year_sine 0.0001 0.3997101 0.6516237 0.9167820
## 3  T_surf_model, PO4_lake, year_sine 0.0003 0.3997302 0.6516152 0.9167728
## 4  T_surf_model, PO4_lake, year_sine 0.0010 0.3998005 0.6515855 0.9167407
## 5  T_surf_model, PO4_lake, year_sine 0.0030 0.4000012 0.6515016 0.9166490
## 6  T_surf_model, PO4_lake, year_sine 0.0100 0.4007028 0.6512080 0.9163287
## 7  T_surf_model, PO4_lake, year_sine 0.0300 0.4026975 0.6503691 0.9154191
## 8  T_surf_model, PO4_lake, year_sine 0.1000 0.4095629 0.6474682 0.9122989
## 9  T_surf_model, PO4_lake, year_sine 0.3000 0.4281289 0.6393865 0.9038847
## 10 T_surf_model, PO4_lake, year_sine 0.5000 0.4450723 0.6315399 0.8961158
## 11 T_surf_model, PO4_lake, year_sine 0.7500 0.4639966 0.6225337 0.8871470
## 12 T_surf_model, PO4_lake, year_sine 1.0000 0.4805999 0.6140992 0.8788528
## 13 T_surf_model, PO4_lake, year_sine 1.5000 0.5078184 0.5986107 0.8639712
## 14 T_surf_model, PO4_lake, year_sine 2.0000 0.5283325 0.5868339 0.8513645
## 15 T_surf_model, PO4_lake, year_sine 3.0000 0.5539831 0.5741003 0.8336031
```

```
## 16 T_surf_model, PO4_lake, year_sine 4.0000 0.5662123 0.5673727 0.8245313
## 17 T_surf_model, PO4_lake, year_sine 6.0000 0.5687217 0.5661643 0.8241121
## 18 T_surf_model, PO4_lake, year_sine 8.0000 0.5565872 0.5770874 0.8376236
```

This “mechanistic” multivariate model is also compared to univariate, seasonal, and multiview predictors of chlorophyll as validation.

```
fname.chl_baselines <- './OUTPUTS/mEDM_chl_baselines.Rdata'

if(!file.exists('./OUTPUTS/mEDM_chl_baselines.Rdata')){
  results_mEDM_multiview_fullfit <-  block_chl_mEDM %>%
    select(-T_surf,-Ptot_lake,-Ptot_epi,-T_air,-year_cosine) %>%
    multiview(lib=c(1,nrow(block_chl_mEDM)),pred=c(1,nrow(block_chl_mEDM)),
              target_column="chl",
              max_lag=3,E=4,
              first_column_time = T)

  results_mEDM_seasonal <- do_mEDM_models(block=block_chl_mEDM,  list( c('year_sine',
'year_cosine') ),target_var='chl',tp=1)$multi_stats[[1]]

  E.univar <- 12
  results_univar <- s_map(data_univar,tau=1,tp=1,E=E.univar)

  save(results_univar,
     results_mEDM_seasonal,
     results_mEDM_multiview_fullfit,
     file=fname.chl_baselines)
}
```

The same analysis is performed for *PO4_surf* as *chl*.

```
block_PO4_epi_mEDM <- data_lake_geneva %>%
    mutate(year_sine=sin(2*pi*yday(date)/365)) %>%
    mutate(year_cosine=cos(2*pi*yday(date)/365)) %>%
    filter(date>="1976-05-08") %>%
    mutate_at(vars(-date),funs(./sd(.,na.rm=TRUE)))

data_univar <- data_lake_geneva %>% filter(date>="1976-05-08") %>% pull(PO4_epi)

fname.PO4_epi_greedy <- './OUTPUTS/mEDM_PO4_epi_greedy.Rdata'

L_candidate_variables <- c('year_sine','chl','rhone','h_mix_model')

if(!file.exists(fname.PO4_epi_greedy)){

  results_mEDM_greedy <- do_mEDM_greedy(block_PO4_epi_mEDM,embed0=c('T_surf_model','P
O4_lake'),L_candidate_variables,target_col="PO4_epi",max_E=6)
  save(results_mEDM_greedy,file=fname.PO4_epi_greedy)
}else{
  load('./OUTPUTS/mEDM_PO4_epi_greedy.Rdata')
}

results_mEDM_greedy %>% select(embedding,theta,rho,mae,rmse) %>% print()
```

```
##                               embedding  theta       rho       mae      rmse
## 1  T_surf_model, PO4_lake, year_sine 0.0000 0.8932136 0.3446796 0.4484646
## 2  T_surf_model, PO4_lake, year_sine 0.0001 0.8932223 0.3446645 0.4484473
## 3  T_surf_model, PO4_lake, year_sine 0.0003 0.8932397 0.3446344 0.4484127
## 4  T_surf_model, PO4_lake, year_sine 0.0010 0.8933006 0.3445291 0.4482916
## 5  T_surf_model, PO4_lake, year_sine 0.0030 0.8934742 0.3442284 0.4479462
## 6  T_surf_model, PO4_lake, year_sine 0.0100 0.8940782 0.3431882 0.4467422
## 7  T_surf_model, PO4_lake, year_sine 0.0300 0.8957724 0.3402750 0.4433469
## 8  T_surf_model, PO4_lake, year_sine 0.1000 0.9013471 0.3305338 0.4319742
## 9  T_surf_model, PO4_lake, year_sine 0.3000 0.9145486 0.3068519 0.4036284
## 10 T_surf_model, PO4_lake, year_sine 0.5000 0.9244568 0.2869655 0.3807466
## 11 T_surf_model, PO4_lake, year_sine 0.7500 0.9334842 0.2673508 0.3583733
## 12 T_surf_model, PO4_lake, year_sine 1.0000 0.9399385 0.2522186 0.3412913
## 13 T_surf_model, PO4_lake, year_sine 1.5000 0.9482204 0.2303637 0.3177501
## 14 T_surf_model, PO4_lake, year_sine 2.0000 0.9530258 0.2154889 0.3030337
## 15 T_surf_model, PO4_lake, year_sine 3.0000 0.9576917 0.1994656 0.2876118
## 16 T_surf_model, PO4_lake, year_sine 4.0000 0.9594321 0.1919100 0.2814219
## 17 T_surf_model, PO4_lake, year_sine 6.0000 0.9595478 0.1889992 0.2808398
## 18 T_surf_model, PO4_lake, year_sine 8.0000 0.9573003 0.1925509 0.2883696
```

As above, the multivariate model is compared to other EDM predictors.

```
fname.PO4_epi_baselines <- './OUTPUTS/mEDM_PO4_epi_baselines.Rdata'

if(!file.exists(fname.PO4_epi_baselines)){
results_mEDM_multiview_fullfit <-  block_PO4_epi_mEDM %>%
        select(-T_surf,-Ptot_lake,-Ptot_epi,-T_air,-year_cosine) %>%
        multiview(lib=c(1,nrow(block_PO4_epi_mEDM)),pred=c(1,nrow(block_PO4_e
pi_mEDM)),
                  target_column="PO4_epi",
                  max_lag=3,E=4,
                  first_column_time = T)

results_mEDM_seasonal <- do_mEDM_models(block=block_PO4_epi_mEDM,  list( c('y
ear_sine','year_cosine') ),target_var='PO4_epi',tp=1)$multi_stats[[1]]

E.univar <- 3
results_univar <- s_map(data_univar,tau=4,tp=4,E=E.univar)

save(results_univar,
     results_mEDM_seasonal,
     results_mEDM_multiview_fullfit,
     file=fname.PO4_epi_baselines)

}
```

State-Dependent Interaction Coefficients

These results are the basis for Figure 2. First, we examine the S-map approximations of the local linear coefficients.

```
L_model_i <- c('T_surf_model','PO4_lake','year_sine')
sim_col <- "chl"

block_model_i <- block_chl_mEDM[,c('date',union(L_model_i,sim_col))]

block_chl_mEDM_raw <- data_lake_geneva %>%
    mutate(year_sine=sin(2*pi*yday(date)/365)) %>%
    mutate(year_cosine=cos(2*pi*yday(date)/365)) %>%
    filter(date>="1976-05-08")

out_s_map <- block_lnlp(block_model_i,theta=6,num_neighbors = 0,method="s-map",column
s = L_model_i,target_column = "chl",stats_only = F,save_smap_coefficients = T,first_c
olumn_time = T)

out_s_map_coeff <- out_s_map$smap_coefficients[[1]] %>%
     rename_at(vars(1:length(L_model_i)),~map_chr(.,function(label_i){
         col_i <- str_extract(label_i,"(?<=c_)[:digit:]+") %>% as.numeric()
         return(paste("c",L_model_i[[col_i]],sep="_"))
     } ))

    bind_cols(block_chl_mEDM_raw,out_s_map_coeff) %>%
        mutate(year = year(date)) %>%
        group_by(year) %>%
        summarise_at(vars(starts_with("c_"),PO4_lake),funs(mean,median)) %>%
        ggplot(aes(x=PO4_lake_median,y=c_PO4_lake_median)) + geom_point() +
        geom_hline(yintercept = 0,lty=2,color="tomato") +
        stat_smooth() +
        labs(title="Median Annual Effect\nof TP on Chl",x="TP (\u03BCg/L)",y="\u2202
Chl / \u2202 TP") +
        theme_bw()
```

Then we examine the coefficients for the multivariate S-map model of DO.

```
L_model_i <- c('PO4_epi','PO4_lake','h_mix_model','T_surf_model','chl','oxydeep')
# L_model_i <- c('PO4_lake','h_mix_model','T_surf_model','chl','oxydeep')
sim_col <- "oxydeep"

block_oxy_mEDM <- data_lake_geneva %>%
    mutate(oxydeep_delta = c(NA,diff(oxydeep))) %>%
    mutate_at(vars(-oxydeep_delta,-date),funs(./sd(.,na.rm=TRUE)))
block_model_i <- block_oxy_mEDM[,c("date",union(L_model_i,sim_col))]

out_s_map <- block_lnlp(block_model_i,theta=6,num_neighbors = 0,method="s-map",column
s = L_model_i,target_column = sim_col,stats_only = F,save_smap_coefficients = T,first
_column_time = T)

## Warning in model$run(): Found overlap between lib and pred. Enabling cross-
## validation with exclusion radius = 0.

out_s_map_coeff <- out_s_map$smap_coefficients[[1]] %>%
    rename_at(vars(1:length(L_model_i)),~map_chr(.,function(label_i){
        col_i <- str_extract(label_i,"(?<=c_)[:digit:]+") %>% as.numeric()
        return(paste("c",L_model_i[[col_i]],sep="_"))
    } ))
```

```r
    bind_cols(data_lake_geneva,out_s_map_coeff) %>%
        filter(month(date) %in% 5:10) %>%
        mutate(year = year(date)) %>%
        group_by(year) %>%
        summarise_at(vars(starts_with("c_"),PO4_lake),funs(mean,median)) %>%
        ggplot(aes(x=PO4_lake_median,y=c_chl_median)) + geom_point() +
        geom_hline(yintercept = 0,lty=2,color="tomato") +
        stat_smooth() +
        labs(title = "Median May-Oct Effect\nof Chl on Bottom DO",x="TP (\u03BCg/L)",
y="\u2202 DO / \u2202 Chl") +
        theme_bw()
```

Iterative forecasts of DO

We now revisit the question of mechanistic predictions of DO. As described above, when DO is included as a possible predictor, the month-to-month forecast skill is very high. Thus, to identify a robust set of drivers for EDM, we examine forecast skill over a longer time-horizon using the newly defined iterative predictor. However, this calculation is computationally intensive so we structure the code so that it can also load results from archived output. The results are the basis for Figure S6.

```r
fname.DO_sim_tp <- "./OUTPUTS/mEDM_DO_sim_tp.Rdata"

if(!file.exists(fname.DO_sim_tp)){
dates_IS <- c("1976-05-08","2012-02-01")

L_models <- list(
     # M1: Figure 1 best model PLUS oxydeep
    c('PO4_epi','PO4_lake','h_mix_model','T_surf_model','T_air','rhone','chl','oxydee
p'),
    # M2: M1 without exogenous physical variables (rho, T_air)
    c('PO4_epi','PO4_lake','h_mix_model','T_surf_model','chl','oxydeep'),
    # M3: M2 without SRP_lake
    c('PO4_lake','h_mix_model','T_surf_model','chl','oxydeep'),
    # M4: M2 without CHL
    c('PO4_epi','PO4_lake','h_mix_model','T_surf_model','oxydeep'),
    # M5: M2 without PO4_epi
    c('PO4_lake','h_mix_model','T_surf_model','chl','oxydeep')
    )

phys_vars <- c("T_surf","h_mix","wind_speed","T_air","rhone","T_surf_model","h_mix_mo
del")
tp_max <- 12

theta_list <- c(0, 1e-04, 3e-04, 0.001, 0.003, 0.01, 0.03, 0.1, 0.3, 0.5,
  0.75, 1, 1.5, 2, 3, 4, 6, 8)

lake_geneva_sim <- data_lake_geneva %>%
    rename(time=date) %>%
```

```
    # mutate(oxydeep_delta = c(NA,diff(oxydeep))) %>%
    mutate_at(vars(phys_vars),funs(lead(.,1))) %>%
    filter(complete.cases(.)) %>%
    filter(time >= dates_IS[1] & time <= dates_IS[2])

I_start_points <- 1:( NROW(lake_geneva_sim) - tp_max)

results_sim_tp_smap <- map_df(L_models,function(L_model_i){

    block_model_i <- lake_geneva_sim[,c('time',L_model_i)]

    results_sim <- map_df(I_start_points,function(i_start){

        block_sim_i <- block_model_i[i_start + 0:tp_max,]

        out_sim_i <- sim_EDM_smap_diff(block_model_i,
                                       block_sim_i,
                                       tp_max,
                                       tp = 1,
                                       # num_neighbors = max(map_dbl(L_models,length)
) + 1,
                                       lib_train = c(1,NROW(block_model_i)),
                                       pred_sim = c(1,NROW(block_model_i)),
                                       sim_col = 'oxydeep', # could set to which colu
mns are NAs
                                       theta = theta_list ,
                                       exclusion_radius = 90
                                       ) %>%
            rename(date=time) %>%
            group_by(type) %>%
            mutate(tp=row_number()-1) %>%
            ungroup()
    }

    )

    results_sim_oxy <- full_join( results_sim %>%
                                      filter(type=='sim') %>%
                                      select(date,tp,oxydeep) %>%
                                      rename(pred=oxydeep) ,
                                  results_sim %>%
                                      filter(type=='true') %>%
                                      select(date,tp,oxydeep) %>%
                                      rename(obs=oxydeep),
                                  by = c("date","tp")) %>%
     mutate(embedding=label_embeddings_parsable(paste(1:length(L_model_i),collapse=",
"),
                                                L_model_i,
                                                dictionary = dictionary_expression
s) %>% as.factor())

}) # map(L_models_)

save(results_sim_tp_smap,file=fname.DO_sim_tp)
}else{
```

```
  load(fname.DO_sim_tp)
}

results_sim_tp_smap %>%
    filter(tp <= 12) %>%
    group_by(tp,embedding) %>%
    summarise(stats=compute_stats(obs,pred) %>% list()) %>%
    mutate(embedding = str_replace_all(embedding,"_model","")) %>%
    unnest(stats) %>%
    ggplot(aes(x=tp,y=rho)) + geom_line(aes(color=embedding),lwd=1) +
    scale_x_continuous(breaks=c(0,3,6,9,12),minor_breaks = 0:12) +
    labs(x='Prediction Time (months)',y='Forecast Skill (\u03C1)',color=NULL) +
    theme_bw() + theme(legend.position = "bottom") +
    scale_color_viridis_d(labels= f_q_exp) +
    guides(color=guide_legend(ncol=1,title=NULL))
```

We then explore the behavior of these 6-month forecasts across summer conditions. As mentioned above we use the simplex predictor instead of S-maps for computational efficiency. These results are used for Figure 3. First, we write a function.

Scenario Modeling

```
EDM_DO_module <- function(block_observed,block_scenario,time_0,DO_0,dt=6){

  L_model <- c('PO4_lake','PO4_epi','h_mix_model','T_surf_model','oxydeep','c
hl')

  tp_max <- dt
  month_start <- month(time_0)

  i_start <- which.min(abs(block_scenario$date - time_0))

  block_sim_i <- block_scenario[i_start + 0:tp_max,]
  block_sim_i[1,'oxydeep'] <- as.numeric(DO_0)

  out_sim_i <-  sim_EDM_simplex_diff(block_train = block_observed %>% rename(
time=date),
                                     block_sim = block_sim_i %>% rename(time=
date),
                                     t_sim = tp_max,
                                     # num_neighbors = 4,
                                     tp = 1,
                                     lib_train = c(1,NROW(block_observed)),
                                     pred_sim = c(1,NROW(block_sim_i)),
                                     sim_col = 'oxydeep', # could set to whic
h columns are NAs
                                     predictor_col=L_model,
                                     exclusion_radius = 0) %>%
    filter(type=="sim") %>%
    tail(1)
```

```
  DO_1 <- out_sim_i$oxydeep

  return(DO_1)
}
```

We are interested in changes in total phosphorus in the lake and atmospheric warming. To simulate effects of atmospheric warming we need to utilize the Simstrat parametric model of lake physics.

```
oxysat = 13 # oxygen saturation at 5∞C [mg/L]
cr_dm = 250 # critical depth of mixed layer to consider lake as undergoing co
mplete mixing
oxyR=10
g = 9.81
alpha = 100e-6
Vbot = 1.4e10
H = 310 - cr_dm
# time_hybrid[1,] = c(1981,5,1,0,0,0);
time_hybrid_0 <- ymd("1981-5-1")

Simstrat_physics_inital <- function(run = FALSE,
                                    file.output = './OUTPUTS/LAKEGENEVA/T_out
.dat',
                                    file.params = "./Inputs/simstrat_LakeGene
va_Deep_Mixing.par"){



  # INPUTS: Simstrat parameter file name
  # OUTPUT: list, [1] "mixing"
  if(run){
    system(paste("simstrat_windows_301.exe",file.params))
  }

  Output_Simstrat_T <- read.csv(file.output,header = FALSE)

  time_mo <- ymd_h("1981-1-1-0") + hours(Output_Simstrat_T[-1,1]*24)
  time_model_1d = seq(round(first(time_mo),"days"),round(last(time_mo),"days"
),by="day");
  # time_mo <- as.Date(Output_Simstrat_T[-1,1],origin="1980-1-1-0-0-0",tz="UT
C")

  z <- -as.numeric(Output_Simstrat_T[1, -1])
  dz <- -diff(z);
  Temp <- as.matrix(Output_Simstrat_T[-1,-1])

  zdeep <- numeric(length(time_mo))
```

```
  Tsurf <-numeric(length(time_mo))

  ## Extraction of surface temperature and thermocline depth
  for(k in 1:length(time_mo)){
    dT <- diff(Temp[k,])
    n2 <- g*alpha*dT/dz
    # [val,ind] = max(n2(1:end));
    ind <- which.max(n2)
    zdeep[k]=z[last(ind)] # thermocline depth estimated based on the max of s
tratification
    Tsurf[k] = Temp[k,NCOL(Temp)]
  }


  # Down-sample (with linear interpolation if slight mismatches) to 1 day.
  zdeep <- approx(x=time_mo,y=zdeep,xout = time_model_1d)$y
  T_surf <- approx(x=time_mo,y=Tsurf,xout = time_model_1d)$y

  h_mix = round(zdeep); # daily mixed-layer depth

  return(data.frame(time=time_model_1d,h_mix=h_mix,T_surf=T_surf))

}
```

First we import archived runs of Simstrat for atmospheric warming scenarios. Code that integrates system calls to Simstrat execution is provided in the Github, but requires independent installation of the Simstrat software.

```
Simstrat_get_warming_scenario <- function(delta_T = 0,
                                          run=F,
                                          initial_output_path = './OUTPUTS/LA
KEGENEVA_SIMSTRAT_1_0/'
                                          ){

  scenario_output_path = paste0(initial_output_path,"Scenario_",delta_T,"degC
_warming/")

  Output_Simstrat_T <- read.csv(paste0(scenario_output_path,"T_out.dat"),head
er = FALSE)

  time_mo <- ymd_h("1981-1-1-0") + hours(Output_Simstrat_T[-1,1]*24)
  time_model_1d = seq(round(first(time_mo),"days"),round(last(time_mo),"days"
),by="day");

  z <- -as.numeric(Output_Simstrat_T[1, -1])
  dz <- -diff(z);
  Temp <- as.matrix(Output_Simstrat_T[-1,-1])
```

```r
  zdeep <- numeric(length(time_mo))
  Tsurf <-numeric(length(time_mo))

  ## Extraction of surface temperature and thermocline depth
  for(k in 1:length(time_mo)){
    dT <- diff(Temp[k,])
    n2 <- g*alpha*dT/dz
    ind <- which.max(n2)
    zdeep[k]=z[last(ind)] # thermocline depth estimated based on the max of s
tratification
    Tsurf[k] = Temp[k,NCOL(Temp)]
  }

  # Down-sample (with linear interpolation if slight mismatches) to 1 day.
  zdeep <- approx(x=time_mo,y=zdeep,xout = time_model_1d)$y
  T_surf <- approx(x=time_mo,y=Tsurf,xout = time_model_1d)$y

  h_mix = round(zdeep); # daily mixed-layer depth

  return(data.frame(time=time_model_1d,h_mix=h_mix,T_surf=T_surf))
}

# rm(list = ls())

data_scenarios_file <- "./INPUTS/EDM_input_data_scenarios.Rdata"

v_T_scenarios = c(0,1,3)
v_PO4_scenarios <- 15:60

if(!file.exists(data_scenarios_file)){
  load( "./INPUTS/EDM_input_data.Rdata")

t_interp <- data_lake_geneva$date

L_input_data_scenarios=vector(mode = "list",length=length(v_T_scenarios))

L_input_data_scenarios[[1]] <- Simstrat_get_warming_scenario(delta_T=v_T_scen
arios[1])
L_input_data_scenarios[[2]] <- Simstrat_get_warming_scenario(delta_T=v_T_scen
arios[2])
L_input_data_scenarios[[3]] <- Simstrat_get_warming_scenario(delta_T=v_T_scen
arios[3])

for(i_scenario in seq(along=L_input_data_scenarios)){
  L_input_data_scenarios[[i_scenario]] <- L_input_data_scenarios[[i_scenario]
] %>%
    rename(date = time) %>%
    mutate(date = ymd(date)) %>%
    mutate(date = cut(date,breaks=c(min(t_interp)-days(30),t_interp),right=TR
UE,labels=(t_interp)) %>% as.Date() ) %>%
```

```
    group_by(date) %>%
    summarise(h_mix_model = max(h_mix,na.rm=T), T_surf_model = mean(T_surf,na
.rm=T))

  L_input_data_scenarios[[i_scenario]] <- full_join(
    data_lake_geneva %>% select(!ends_with("_model")),
    L_input_data_scenarios[[i_scenario]],
    by = "date"
  )
}

save(L_input_data_scenarios,file=data_scenarios_file)
}

load(data_scenarios_file)
```

With the Simstrat outputs for each temperature scenario, we can now iterate through the combined temperature and nutrient scenarios, calling the EDM simulation function each time.

```
load("./INPUTS/EDM_input_data.Rdata")

v_PO4_scenarios <- seq(15,60,by=1 )

block_model <- data_lake_geneva %>%
  mutate(year_sine=sin(2*pi*yday(date)/365)) %>%
  mutate(year_cosine=cos(2*pi*yday(date)/365)) %>%
  filter(date > ymd("1981-02-01"))

block_in <- data_lake_geneva %>%
  mutate_at(c('h_mix_model','T_surf_model'),funs(lead(.,1))) %>%
  # filter(year(date) <= 2013) %>%
  filter(date > ymd("1981-02-01"))

tp_max <- 6

v_T_scenarios = c(0,1,3)
may_oxydeep <- c(4.5,6,7.5)
# may_oxydeep <- 4.5

results_oxydeep_scen_exp <- map_dfr(seq(along=v_T_scenarios),function(i_T) {

  map_dfr(seq(along=v_PO4_scenarios),function(i_PO4){

    T_scenario <- v_T_scenarios[i_T]
    PO4_scenario <- v_PO4_scenarios[i_PO4]

    # Generate BGC forcing data from CHL and PO4_epi S-map models

    # Begin with block that has h_mix_model and T_surf_model based on i_T
```

```
    block_sim_i <- L_input_data_scenarios[[i_T]] %>%
      mutate(year_sine=sin(2*pi*yday(date)/365)) %>%
      mutate(year_cosine=cos(2*pi*yday(date)/365)) %>%
      select(names(block_model)) %>%
      filter(date > ymd("1981-01-01"))

    # replace PO4_lake with the scenario
    block_sim_i$PO4_lake <- PO4_scenario

    # run chl model with scenario forcing and replace in block_sim
    L_model_chl <- c('T_surf_model','PO4_lake','year_sine')

    # sim_EDM_simplex
    out_sim_chl_i <- sim_EDM_smap(block_model %>% rename(time=date),
                                  # out_sim_i <- sim_EDM_smap_diff(block_mode
l_i,
                                  block_sim_i %>% rename(time=date),
                                  NROW(block_sim_i),
                                  tp = 1,
                                  lib_train = c(1,NROW(block_model)),
                                  pred_sim = c(1,NROW(block_sim_i)),
                                  sim_col = "chl",
                                  predictor_col=L_model_chl,
                                  theta = c(0,.1,.5,1,2,3,4,5,6,7,8),
                                  exclusion_radius = 0) %>%
      rename(date=time) %>%
      filter(type=='sim') %>%
      select(date,chl)

    block_sim_i <- left_join(block_sim_i %>% select(-chl),
                             out_sim_chl_i,by="date") %>%
      select(names(block_model))

    # run PO4_epi with scenario forcing and replace in block_sim
    # L_model_PO4_epi <- c('T_surf_model','PO4_lake','year_sine')
    L_model_PO4_epi <- c('T_surf_model','PO4_lake','year_sine','PO4_epi')

    out_sim_PO4_epi_i <- sim_EDM_smap(block_model %>% rename(time=date),
                                      # out_sim_i <- sim_EDM_smap_diff(block_
model_i,
                                      block_sim_i %>% rename(time=date),
                                      NROW(block_sim_i),
                                      tp = 1,
                                      lib_train = c(1,NROW(block_model)),
                                      pred_sim = c(1,NROW(block_sim_i)),
                                      sim_col = "PO4_epi", # could set to whi
ch columns are NAs
                                      predictor_col=L_model_PO4_epi,
                                      theta = c(0,.1,.5,1,2,3,4,5,6,7,8),
                                      exclusion_radius = 0) %>%
```

```
      rename(date=time) %>%
      filter(type=='sim') %>%
      select(date,PO4_epi)

    block_sim_i <- left_join(block_sim_i %>% select(-PO4_epi),
                             out_sim_PO4_epi_i,by="date") %>%
      select(names(block_model))

    # adjust time index of physical drivers so current values are used for pr
ediction rather than 1-step lags.

    block_sim_i <- block_sim_i[,names(block_in)] %>%
      mutate_at(c('h_mix_model','T_surf_model'),funs(lead(.,1))) %>%
      filter(row_number() > 1)

    # run EDM simulation.

    I_start_points <- 1:( NROW(block_sim_i) - tp_max)
    I_start_points <- which(month(block_sim_i$date) == 5)


    temp <- map_df(I_start_points,function(i_start){
      map_df(may_oxydeep,function(may_oxydeep_i){
        oxydeep <- EDM_DO_module(block_in,block_sim_i,time_0 = block_sim_i$da
te[i_start],DO_0=may_oxydeep_i,dt=6)
       return(data.frame(T_scenario=T_scenario,PO4_scenario=PO4_scenario,DO_0
=may_oxydeep_i,DO=oxydeep))
      }) # map may_oxydeep_i
    } ) # map i_start
    # return(temp %>% mutate(T_scenario=T_scenario,PO4_scenario=PO4_scenario)
)
    return(temp)

  }) # map i_PO4
}) # for i_T

save(results_oxydeep_scen_exp,file="./OUTPUTS/RESULTS_figure_3.Rdata")

# load("./OUTPUTS/RESULTS_figure_3.Rdata",envir=E_fig3)
load("./OUTPUTS/RESULTS_figure_3.Rdata")

results_oxydeep_scen_exp %>%
  group_by(DO_0) %>%
  mutate(delta_oxydeep= (DO-DO_0) / 184) %>%
    ggplot(aes(color=as.factor(T_scenario),y=delta_oxydeep,x=PO4_scenario)) +
    geom_point(alpha=.4) + stat_smooth(method = 'loess',span=0.4) +
    xlim(c(15,60)) +
    facet_wrap(~DO_0)

## `geom_smooth()` using formula 'y ~ x'
```

Hybrid Modeling

Having selected a set of embedding variables to define our multivariate EDM model for DO, we can now write the EDM predictor module that goes inside of the hybrid model. It is a function that calls in the training data, the data representing the simulation scenario, the time we are initializing the forecast, the initial condition of oxygen, and the length of time (in months) to perform the iterative forecasts.

```
EDM_DO_module <- function(block_observed,block_scenario,time_0,DO_0,dt=6){

  L_model <- c('PO4_lake','PO4_epi','h_mix_model','T_surf_model','oxydeep','chl')

  tp_max <- dt
  month_start <- month(time_0)

  i_start <- which.min(abs(block_scenario$date - time_0))

  block_sim_i <- block_scenario[i_start + 0:tp_max,]
  block_sim_i[1,'oxydeep'] <- as.numeric(DO_0)

  out_sim_i <-  sim_EDM_simplex_diff(block_train = block_observed %>% rename(time=dat
e),
                                     block_sim = block_sim_i %>% rename(time=date),
                                     t_sim = tp_max,
                                     # num_neighbors = 4,
                                     tp = 1,
                                     lib_train = c(1,NROW(block_observed)),
                                     pred_sim = c(1,NROW(block_sim_i)),
                                     sim_col = 'oxydeep', # could set to which column
s are NAs
                                     predictor_col=L_model,
                                     exclusion_radius = 0) %>%
    filter(type=="sim") %>%
    tail(1)

  DO_1 <- out_sim_i$oxydeep

  return(DO_1)
}
```

The rest of the hybrid model structure is now described. This requires several physical parameters for the two-box model as well as a function to process Simstrat outputs to drive the EDM module and 2-box model. The function includes a suggestion of how to automatically run Simstrat embedded within the code on a machine that already has the executable in the local

directory.

```
oxysat = 13 # oxygen saturation at 5∞C [mg/L]
cr_dm = 250 # critical depth of mixed layer to consider lake as undergoing complete m
ixing
oxyR=10
g = 9.81
alpha = 100e-6
Vbot = 1.4e10
H = 310 - cr_dm
# time_hybrid[1,] = c(1981,5,1,0,0,0);
time_hybrid_0 <- ymd("1981-5-1")

Simstrat_physics_inital <- function(run = FALSE,
                                    file.output = './Outputs/LakeGeneva/T_out.dat',
                                    file.params = "./Inputs/simstrat_LakeGeneva_Deep_
Mixing.par"){

  if(run){
    system(paste("simstrat_windows.exe",file.params))
  }

  Output_Simstrat_T <- read.csv(file.output,header = FALSE)

  time_mo <- ymd_h("1981-1-1-0") + hours(Output_Simstrat_T[-1,1]*24)
  time_model_1d = seq(round(first(time_mo),"days"),round(last(time_mo),"days"),by="da
y");
  # time_mo <- as.Date(Output_Simstrat_T[-1,1],origin="1980-1-1-0-0-0",tz="UTC")

  z <- -as.numeric(Output_Simstrat_T[1, -1])
  dz <- -diff(z);
  Temp <- as.matrix(Output_Simstrat_T[-1,-1])

  zdeep <- numeric(length(time_mo))
  Tsurf <-numeric(length(time_mo))

  ## Extraction of surface temperature and thermocline depth
  for(k in 1:length(time_mo)){
    dT <- diff(Temp[k,])
    n2 <- g*alpha*dT/dz
    # [val,ind] = max(n2(1:end));
    ind <- which.max(n2)
    zdeep[k]=z[last(ind)] # thermocline depth estimated based on the max of stratific
ation
    Tsurf[k] = Temp[k,NCOL(Temp)]
  }

  # Down-sample (with linear interpolation if slight mismatches) to 1 day.
  zdeep <- approx(x=time_mo,y=zdeep,xout = time_model_1d)$y
  T_surf <- approx(x=time_mo,y=Tsurf,xout = time_model_1d)$y

  h_mix = round(zdeep); # daily mixed-layer depth

  return(data.frame(time=time_model_1d,h_mix=h_mix,T_surf=T_surf))
}
```

```
hybrid_model_v1 <- function(
  block_observed,
  block_scenario = block_observed,
  t0="1981-5-15",    # a "yyyy-mm-dd" string or POSIX date
  DO_0=9.8958,
  n_steps=70,
  dt=6              # in months
){
  if(is.character(t0)){
    t0 = ymd(t0)
  }

  df_hybrid_out <- data.frame(time = seq(t0,by=paste(dt,"months"),length.out = n_step
s),
                              oxydeep = NA)
  df_hybrid_out$oxydeep[1] = DO_0

  # Perform initializing run of Simstrat to get lake physics over model duration
  Simstrat_out <- Simstrat_physics_inital()
  load(file= "./INPUTS/Q_rhone.Rdata")

  Rhone <- approx(x=Rhone$time,y=Rhone$Q,xout=Simstrat_out$time,method="linear",rule=
2) %>%
    data.frame() %>% rename(time=x,Q_rhone=y)

  k=1

  while(k<n_steps){
    k=k+1

    time_k = df_hybrid_out$time[k-1] # time at start of interval k
    month = month(time_k)
    yy = year(time_k)

    # time_hybrid(k,:) = ([1981,5+6*(k-1),1,0,0,0]);
    # month = time_hybrid(k,2);

    # DO_k_0 = round(df_hybrid_out$oxydeep[k-1],1); #initial DO in kth interval
    DO_k_0 = df_hybrid_out$oxydeep[k-1]

    # # check if model interval is over summer
    if(month %in% 5:10){
      # generate block_scenario
      # load(paste0('scenario.C_T=0.PO4_lake=',num2str(TP),'.Rdata'))

      # run EDM module
      DO_k <- EDM_DO_module(time_0=time_k,
                            block_observed=block_observed,
                            block_scenario=block_scenario,
                            DO_0=DO_k_0,
                            dt=dt)

      df_hybrid_out$oxydeep[k] = DO_k
```

```
    }else{

      # if the model is over winter, invoke parametric relationships described in Sch
wefel et al. 2016 for 2-box oxygen model

      deep_mixing <- block_scenario %>%
        filter(date >= time_k, date <= time_k + months(dt)) %>%
        pull(h_mix_model) %>%
        max()

      # deep_mixing = max(Simstrat_out$h_mix[I_year]);

      if(deep_mixing < cr_dm){
        # run EDM module over winter period without complete mixing

        DO_k <- EDM_DO_module(time_0=time_k-months(dt),
                              block_observed=block_observed,
                              block_scenario=block_scenario,
                              DO_0=DO_k_0,
                              dt=dt)

        # account for Rhone river inputs when there is no mixing mixing using paramet
ric structure

        # intQ = Rhone %>%
        #   filter(time >= time_k, time <= time_k + months(dt)) %>%
        #   pull(Q_rhone)
        # Qtot = mean(intQ)*length(intQ)*86400; # multiply integrated flux by elapsed
time in seconds
        # DO_k = Qtot/Vbot*oxyR +(Vbot-Qtot)/Vbot*DO_k;

        df_hybrid_out$oxydeep[k]=DO_k;

      }else{
        h1 = deep_mixing-cr_dm;
        h2 = 310-deep_mixing;
        DO_k = h1/H*oxysat + h2/H*DO_k_0

        DO_k =  EDM_DO_module(time_0=time_k-months(dt),
                              block_observed=block_observed,
                              block_scenario=block_scenario,
                              DO_0=DO_k,
                              dt=dt)

        df_hybrid_out$oxydeep[k] = DO_k
      }

    }

    if(df_hybrid_out$oxydeep[k] < 0){
      df_hybrid_out$oxydeep[k] = 0
    }

  } # while(k)
```

```
  return(df_hybrid_out)
}
```

Historical predictions

The hybrid model is first run retrospectively. These results are used for Figure 4a

```
E_fig4 <- new.env()

load("./INPUTS/EDM_input_data.Rdata")

block_in <- data_lake_geneva %>%
  mutate_at(c('h_mix_model','T_surf_model'),funs(lead(.,1))) %>%
  filter(date > ymd("1981-01-01"))


block_sim <- data_lake_geneva %>%
  mutate(year_sine=sin(2*pi*yday(date)/365)) %>%
  mutate(year_cosine=cos(2*pi*yday(date)/365)) %>%
  select(names(block_in)) %>%
  filter(date > ymd("1981-01-01"))

# run chl model with scenario forcing and replace in block_sim
L_model_chl <- c('T_surf_model','PO4_lake','year_sine')

# sim_EDM_simplex
out_sim_chl <- sim_EDM_smap(block_sim %>% rename(time=date),
                            block_sim %>% rename(time=date),
                            NROW(block_sim),
                            tp = 1,
                            lib_train = c(1,NROW(block_sim)),
                            pred_sim = c(1,NROW(block_sim)),
                            sim_col = "chl",
                            predictor_col=L_model_chl,
                            theta = c(0,.1,.5,1,2,3,4,5,6,7,8),
                            exclusion_radius = 0) %>%
  rename(date=time) %>%
  filter(type=='sim') %>%
  select(date,chl)

block_sim <- left_join(block_sim %>% select(-chl),
                       out_sim_chl,by="date") %>%
  select(names(block_in))

# run PO4_epi with scenario forcing and replace in block_sim
L_model_PO4_epi <- c('T_surf_model','PO4_lake','year_sine','PO4_epi')

out_sim_PO4_epi <- sim_EDM_smap(block_sim %>% rename(time=date),
                                block_sim %>% rename(time=date),
                                NROW(block_sim),
                                tp = 1,
                                lib_train = c(1,NROW(block_sim)),
                                pred_sim = c(1,NROW(block_sim)),
                                sim_col = "PO4_epi",
                                predictor_col=L_model_PO4_epi,
```

```
                                  theta = c(0,.1,.5,1,2,3,4,5,6,7,8),
                                  exclusion_radius = 0) %>%
  rename(date=time) %>%
  filter(type=='sim') %>%
  select(date,PO4_epi)

block_sim <- left_join(block_sim %>% select(-PO4_epi),
                       out_sim_PO4_epi,by="date") %>%
  select(names(block_in))

# adjust time index of physical drivers so current values are used for prediction rat
her than 1-step lags.

block_sim <- block_sim[,names(block_in)] %>%
  mutate_at(c('h_mix_model','T_surf_model'),funs(lead(.,1))) %>%
  filter(row_number() > 1)

temp = hybrid_model_v1(block_observed = block_in,block_scenario = block_sim,n_steps=7
2,dt=6)

df_hybrid <- temp %>% mutate(year = year(time),month = month(time), day = day(time))
%>% select(year,month,day,oxydeep) %>% mutate(data="Hybrid")
```

Scenario Exploration

To perform the long-term exploration of future scenarios with the hybrid model, first we import archived runs of Simstrat for atmospheric warming scenarios. Code that integrates system calls to Simstrat execution is provided in the Github, but requires independent installation of the Simstrat software.

```
Simstrat_get_warming_scenario <- function(delta_T = 0,
                                          run=F,
                                          initial_output_path = './Outputs/LAKEGENEVA
_SIMSTRAT_1_0/'
                                          ){

  scenario_output_path = paste0(initial_output_path,"Scenario_",delta_T,"degC_warming
/")

  Output_Simstrat_T <- read.csv(paste0(scenario_output_path,"T_out.dat"),header = FAL
SE)

  time_mo <- ymd_h("1981-1-1-0") + hours(Output_Simstrat_T[-1,1]*24)
  time_model_1d = seq(round(first(time_mo),"days"),round(last(time_mo),"days"),by="da
y");

  z <- -as.numeric(Output_Simstrat_T[1, -1])
  dz <- -diff(z);
  Temp <- as.matrix(Output_Simstrat_T[-1,-1])

  zdeep <- numeric(length(time_mo))
```

```
  Tsurf <-numeric(length(time_mo))

  ## Extraction of surface temperature and thermocline depth
  for(k in 1:length(time_mo)){
    dT <- diff(Temp[k,])
    n2 <- g*alpha*dT/dz
    ind <- which.max(n2)
    zdeep[k]=z[last(ind)] # thermocline depth estimated based on the max of stratific
ation
    Tsurf[k] = Temp[k,NCOL(Temp)]
  }

  # Down-sample (with linear interpolation if slight mismatches) to 1 day.
  zdeep <- approx(x=time_mo,y=zdeep,xout = time_model_1d)$y
  T_surf <- approx(x=time_mo,y=Tsurf,xout = time_model_1d)$y

  h_mix = round(zdeep); # daily mixed-layer depth

  return(data.frame(time=time_model_1d,h_mix=h_mix,T_surf=T_surf))
}

# rm(list = ls())

data_scenarios_file <- "./INPUTS/EDM_input_data_scenarios.Rdata"

v_T_scenarios = c(0,1,3)
v_PO4_scenarios <- 15:60

if(!file.exists(data_scenarios_file)){
  load( "./INPUTS/EDM_input_data.Rdata")

t_interp <- data_lake_geneva$date

L_input_data_scenarios=vector(mode = "list",length=length(v_T_scenarios))

L_input_data_scenarios[[1]] <- Simstrat_get_warming_scenario(delta_T=v_T_scenarios[1]
)
L_input_data_scenarios[[2]] <- Simstrat_get_warming_scenario(delta_T=v_T_scenarios[2]
)
L_input_data_scenarios[[3]] <- Simstrat_get_warming_scenario(delta_T=v_T_scenarios[3]
)

for(i_scenario in seq(along=L_input_data_scenarios)){
  L_input_data_scenarios[[i_scenario]] <- L_input_data_scenarios[[i_scenario]] %>%
    rename(date = time) %>%
    mutate(date = ymd(date)) %>%
    mutate(date = cut(date,breaks=c(min(t_interp)-days(30),t_interp),right=TRUE,label
s=(t_interp)) %>% as.Date() ) %>%
    group_by(date) %>%
    summarise(h_mix_model = max(h_mix,na.rm=T), T_surf_model = mean(T_surf,na.rm=T))

  L_input_data_scenarios[[i_scenario]] <- full_join(
    data_lake_geneva %>% select(!ends_with("_model")),
    L_input_data_scenarios[[i_scenario]],
    by = "date"
```

```
  )
}

save(L_input_data_scenarios,file=data_scenarios_file)
}

load(data_scenarios_file)
```

With the Simstrat outputs for each temperature scenario, we can now iterate through the combined temperature and nutrient scenarios, calling the hybrid model function each time.

```
v_PO4_scenarios <- seq(15,60,by=1 )

block_model <- data_lake_geneva %>%
  mutate(year_sine=sin(2*pi*yday(date)/365)) %>%
  mutate(year_cosine=cos(2*pi*yday(date)/365)) %>%
  filter(date > ymd("1981-02-01"))

block_in <- data_lake_geneva %>%
  mutate_at(c('h_mix_model','T_surf_model'),funs(lead(.,1))) %>%
  # filter(year(date) <= 2013) %>%
  filter(date > ymd("1981-02-01"))

results_scenarios <- map_dfr(seq(along=v_T_scenarios),function(i_T) {

  map_dfr(seq(along=v_PO4_scenarios),function(i_PO4){

    T_scenario <- v_T_scenarios[i_T]
    PO4_scenario <- v_PO4_scenarios[i_PO4]

    # Generate BGC forcing data from CHL and PO4_epi S-map models
    # Begin with block that has h_mix_model and T_surf_model based on i_T
    block_sim_i <- L_input_data_scenarios[[i_T]] %>%
      mutate(year_sine=sin(2*pi*yday(date)/365)) %>%
      mutate(year_cosine=cos(2*pi*yday(date)/365)) %>%
      select(names(block_model)) %>%
      filter(date > ymd("1981-01-01"))

    # replace PO4_lake with the scenario
    block_sim_i$PO4_lake <- PO4_scenario

    # run chl model with scenario forcing and replace in block_sim
    L_model_chl <- c('T_surf_model','PO4_lake','year_sine')

    # sim_EDM_simplex
    out_sim_chl_i <- sim_EDM_smap(block_model %>% rename(time=date),
                                  # out_sim_i <- sim_EDM_smap_diff(block_model_i,
                                  block_sim_i %>% rename(time=date),
                                  NROW(block_sim_i),
                                  tp = 1,
                                  lib_train = c(1,NROW(block_model)),
                                  pred_sim = c(1,NROW(block_sim_i)),
                                  sim_col = "chl",
                                  predictor_col=L_model_chl,
```

```
                                  theta = c(0,.1,.5,1,2,3,4,5,6,7,8),
                                  exclusion_radius = 0) %>%
      rename(date=time) %>%
      filter(type=='sim') %>%
      select(date,chl)

    block_sim_i <- left_join(block_sim_i %>% select(-chl),
                             out_sim_chl_i,by="date") %>%
      select(names(block_model))

    # run PO4_epi with scenario forcing and replace in block_sim
    # L_model_PO4_epi <- c('T_surf_model','PO4_lake','year_sine')
    L_model_PO4_epi <- c('T_surf_model','PO4_lake','year_sine','PO4_epi')

    out_sim_PO4_epi_i <- sim_EDM_smap(block_model %>% rename(time=date),
                                      # out_sim_i <- sim_EDM_smap_diff(block_model_i,
                                      block_sim_i %>% rename(time=date),
                                      NROW(block_sim_i),
                                      tp = 1,
                                      lib_train = c(1,NROW(block_model)),
                                      pred_sim = c(1,NROW(block_sim_i)),
                                      sim_col = "PO4_epi", # could set to which colum
ns are NAs
                                      predictor_col=L_model_PO4_epi,
                                      theta = c(0,.1,.5,1,2,3,4,5,6,7,8),
                                      exclusion_radius = 0) %>%
      rename(date=time) %>%
      filter(type=='sim') %>%
      select(date,PO4_epi)

    block_sim_i <- left_join(block_sim_i %>% select(-PO4_epi),
                             out_sim_PO4_epi_i,by="date") %>%
      select(names(block_model))

    # adjust time index of physical drivers so current values are used for prediction
rather than 1-step lags.

    block_sim_i <- block_sim_i[,names(block_in)] %>%
      mutate_at(c('h_mix_model','T_surf_model'),funs(lead(.,1))) %>%
      filter(row_number() > 1)

    # run hybrid model.
    temp = hybrid_model_v1(block_observed = block_in,block_scenario = block_sim_i,n_s
teps=71,dt=6)

    return(temp %>% mutate(T_scenario=T_scenario,PO4_scenario=PO4_scenario))



  }) # map i_PO4
}) # for i_T

save(results_scenarios,file="./OUTPUTS/hybrid_model_scenarios.Rdata")
```

```
results_scenarios %>%
  group_by(T_scenario,PO4_scenario) %>%
  summarise(perc_hypox = mean(oxydeep < 4)) %>%
  ggplot(aes(x=PO4_scenario,y=perc_hypox,color=factor(T_scenario))) + geom_point(pch=
3) +
  theme_bw() +
  stat_smooth(method="loess",span=0.65) +
  scale_color_manual(values=c("green","blue","red"),limits=c(0,1,3)) +
  ylim(0,1) + xlim(0,60)
```

Driver	n	Cross-map Skill (ρ)
h_{mix} (Simstrat)	524	0.574
T_{atm}	524	0.553
T_{surf}	526	0.512
Q	524	0.403
h_{mix}	528	0.375
SRP_{surf}	524	0.308
TP_{surf}	524	0.301
W	524	0.201
SRP_{lake}	524	0.187
TP_{lake}	524	0.176
Chl	478	0.135

Table S1: Cross-map skill indicating the strength of causal effects of purported drivers on month-to-month changes in DO_B. Here cross-map skill is quantified by Pearson's ρ between observed value of the driver and the predicted value cross-mapped by DO_B (13). Variables as ranked by CCM ρ are depth of mixed layer (h_{mix}), air temperature (T_{atm}), lake surface temperature (T_{surf}), Rhone River discharge (Q), surface averaged soluble reactive phosphorus (SRP_{surf}), surface averaged total phosphorus (TP_{surf}), wind speed (W), lake averaged total phosphorus (TP_{lake}), lake averaged soluble reactive phosphorus (SRP_{lake}), and chlorophyll-a (chl). Note that the monthly averaged values of mixed layer depth (h_{mix}) output from the Simstrat physical model had stronger (higher ρ) CCM results than the direct monthly measurement. The number of predictions, n, is also given for each cross-map test, and all values of ρ for the tested drivers are statistically significant at the $p < 0.05$ under Fischer's Z-transform.

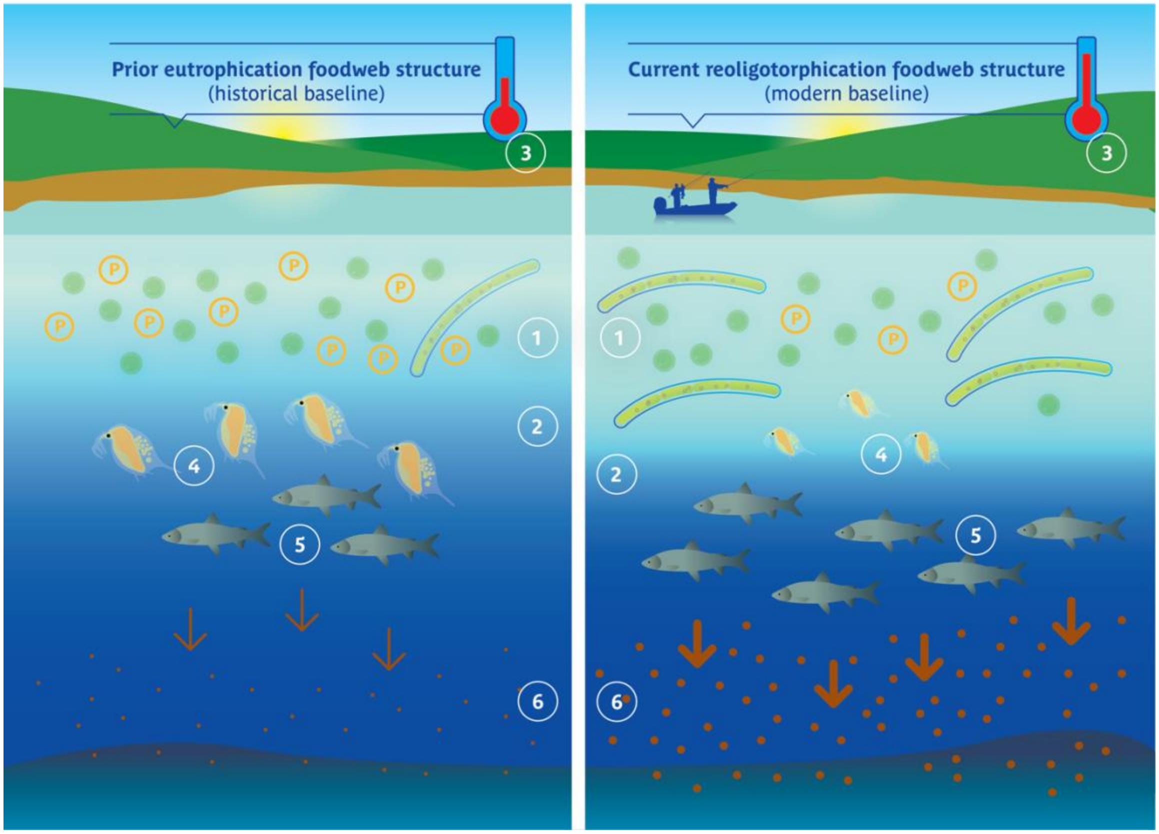

Figure S1: Illustration of the food web rearrangement in Lake Geneva documented in previous studies prior and after re-oligotrophication (1960-2015). Most significant to this study, the food web rearrangement includes changes in the pelagic food web functioning that controls organic matter exports that ultimately condition the rate of deep oxygen consumption. (1) Prior to re-oligotrophication in the 1990s, the phytoplankton of Lake Geneva was dominated by small pelagic algae (mostly diatoms, *Cyclotella* spp. (14). Since re-oligotrophication, *Mougeotia gracillima*, a non-toxic filamentous macroalgae, has increasingly dominated the phytoplankton composition (15). *M. gracillima* is considered as inedible for most zooplankton species (16). *M. gracillima* favors mesotrophic conditions (i.e. ~ 20 µg TP L^{-1}, (17, 18)) and (2) dominance may also be supported by the increasingly strong and deep thermocline resulting from (3) increasing atmospheric temperatures. (4) At the same time, the main zooplankton grazer, *Daphnia* spp. has decreased in size (19) and abundance (9, 19). (5) The main zooplanktivorous fish,

Coregonus lavaretus, has undergone large increases in abundance (20). (6) Finally, in Lake Geneva, exported organic matter, with an export ratio of ~34%, is mostly autochthonous (21). The consequence of all these changes are inferred to be a decoupling of surface chlorophyll from nutrient concentrations, and a greater fraction of primary carbon exported to deep water and sediments.

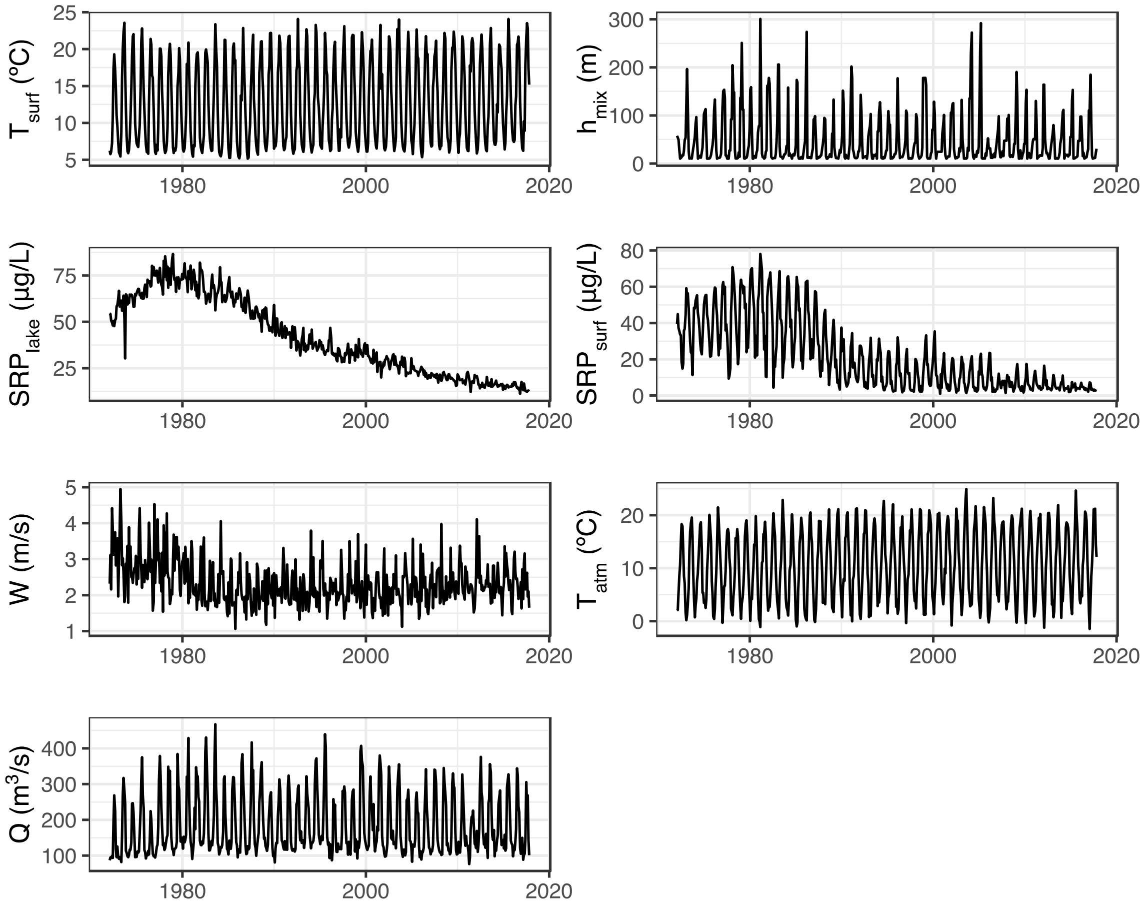

Figure S2: Time series of study variables not shown in main text Figure 1. From left to right, top to bottom, variables are lake surface temperature (T_{surf}), mixed layer depth (h_{mix}), lake averaged soluble reactive phosphorus (SRP_{lake}), surface averaged soluble reactive phosphorus (SRP_{surf}), wind speed (W), air temperature (T_{atm}), and Rhone River discharge (Q).

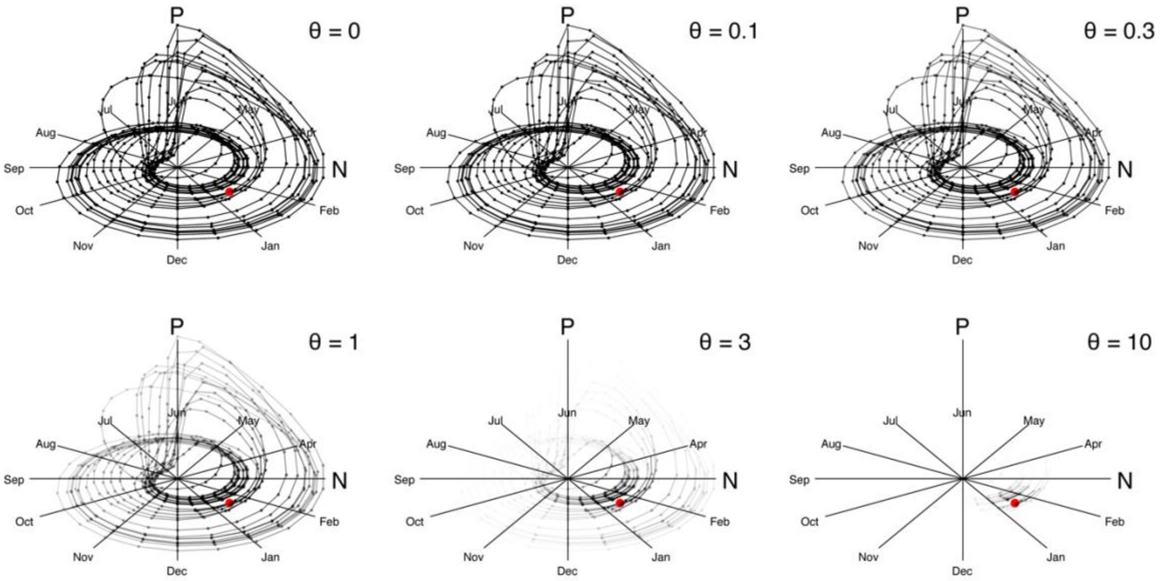

Figure S3: Didactic illustration of the weighting function used in multivariate S-map regression, demonstrated on synthetic data. Here, the data are generated from a simple nutrient-phytoplankton (NP) system of differential equations so the attractors can be reasonably reproduced on the 2-dimensional page using just three coordinate variables (season, nutrients, N, and phytoplankton concentration, P). For S-map regression, observations in the state space are weighted based on normalized Euclidian distance to the forecast target (red circle). Explicitly, $w_i = \exp\left(-\theta d_i/\bar{d}\right)$, where d_i is the Euclidian distance to the target and $\bar{d}$ is the average distance of observations in the training set. The degree of weighting is controlled by the nonlinear parameter, θ, which controls the scaling of the exponentially decaying weight function. When θ=0, all weights are equal, but as θ increases to large values (shown here up to θ=10), the regression is increasingly sensitive to just the immediate neighborhood of the target. In this way, the S-map regression approximates the local linear dynamics on the trajectories in state-space, i.e. the system Jacobian. Although the example we show here was designed to fit in 3-dimensions, the weighting on Euclidian distance works just as well in higher dimensions (e.g. the S-map analysis in main text Figure 1D includes up to 7 dimensions).

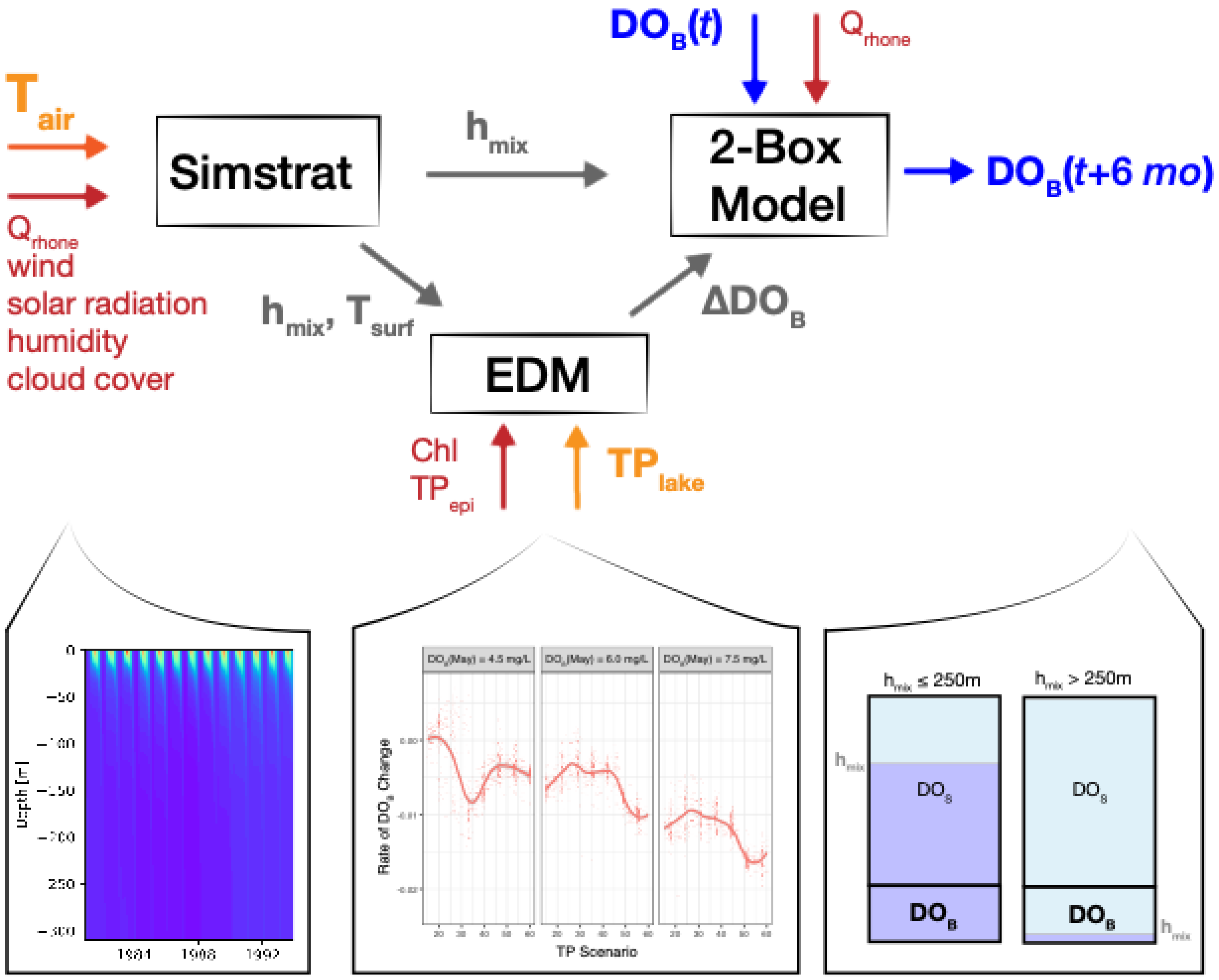

Figure S4: structure of the hybrid model. The hybrid model has three connected components: a 1-D hydrodynamic model of the lake (Simstrat), a 2-box model of dissolved oxygen, and an EDM model simulating oxygen consumption in deep water. The change in oxygen over a 6-month period is treated as the sum of oxygen supply from the surface through mixing, seasonal inputs of oxygenated river water, and consumption at depth due to biogeochemical activity. Anthropogenic forcing is split into 2 scenarios (orange arrows), increases in air temperature related to climate change that directly affect the Simstrat component and another for change in the watershed use that modify the nutrient loading (i.e., re-oligotrophication) that directly affect the EDM component. The climate change scenario is used to force the equation-based model

(Simstrat) which estimates the evolution of the lake thermal structure and outputs mixed-layer height (h_{mix}) based on the depth of the thermocline (grey arrow). This drives the simple 2-box model. When h_{mix} exceeds 250m, increase in oxygen from mixing is estimated based on the fraction of the hypolimnion waters above h_{mix}. Historically, this only occurs in winter months. In winter months without deep mixing, water discharged from the Rhone river will sink to the bottom. With or without mixing, the estimated lake thermal structure (h_{mix}) as well as the re-oligotrophication scenario then serve as forcing parameters for the equation free model (EDM) estimating the rate of DO_B change over 6-months, encapsulated in Figure 3.

Chlorophyll

(A)

Variable	tp	ρ
sin(ν)	−5	0.8235
T_{surf} (SIMStrat)	−5	0.8057
T_{atm}	−5	0.7821
T_{surf}	−5	0.776
Q	−5	0.6601
h_{mix} (SIMStrat)	−2	0.623
SRP_{surf}	−5	0.61
TP_{surf}	−5	0.5904
h_{mix}	−2	0.5715
SRP_{lake}	−5	0.537
TP_{lake}	−5	0.5312
W	−4	0.2059
DO	−1	0.02254

(B)

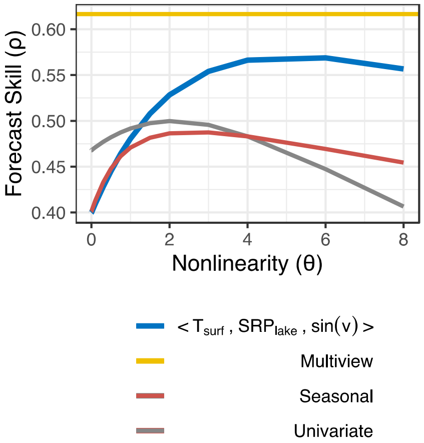

TP (Surface)

(C)

Variable	tp	ρ
sin(ν)	−5	0.8235
T_{surf} (SIMStrat)	−5	0.8057
T_{atm}	−5	0.7821
T_{surf}	−5	0.776
Q	−5	0.6601
h_{mix} (SIMStrat)	−2	0.623
SRP_{surf}	−5	0.61
TP_{surf}	−5	0.5904
h_{mix}	−2	0.5715
SRP_{lake}	−5	0.537
TP_{lake}	−5	0.5312
W	−4	0.2059
DO	−1	0.02254

(D)

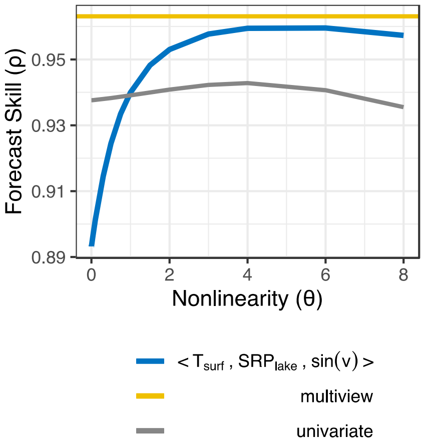

Figure S5: Identifying mechanistic embeddings for other dynamic biogeochemistry variables. First, convergent cross-mapping is used to distinguish possible drivers from CHL (top) and TP_{surf} (bottom). Variables are the same as in Table S1, with the addition of a seasonal index, sin(ν), where $\nu = 2\pi(\textit{day of year})/365$. To generate a minimum dimensional explicit embedding for predicting CHL and TP_{surf} dynamics under management scenarios, we then use a greedy search approach on the most relevant drivers. In each case we begin with the two drivers at the core of management questions, lake temperature in the epilimnion $T_{surf}(t)$ output from the Simstrat model and total phosphorus across all depths $TP_{lake}(t)$, then try adding other variables with significant evidence of causal association based on time-lag CCM analysis (A,C). To predict monthly dynamics of CHL, we find that adding the seasonal cycle, sin(v) where v is the

celestial angle of the Earth from perigee tracking insolation I(t) to the S-map regression on the drivers $T_{surf}(t)$ and $TP_{lake}(t)$, improves forecasting, but additional drivers do not. To predict monthly dynamics of TP_{surf}, we find the same. These models can be roughly interpreted as nonstationary seasonal cycles where oscillations depend on nutrient loading and temperature. In both cases, these embeddings out-perform univariate and simple stationary seasonal models (B,D). The multiview forecast skill (22) is also included as a useful comparison; multiview is a model averaging procedure for obtaining highly accurate EDM predictions in exchange for clear (mechanistic) interpretation. The multiview forecast skill (which does not depend on a nonlinear tuning parameter) can be thought of as an estimate on the upper bound predictability possible from low-dimensional nonlinear analysis of these variables. Note that for predicting $TP_{surf}(t + 1mo)$, a small additional improvement in forecast skill is possible by also including the 0th univariate time lag, i.e. $TP_{surf}(t)$.

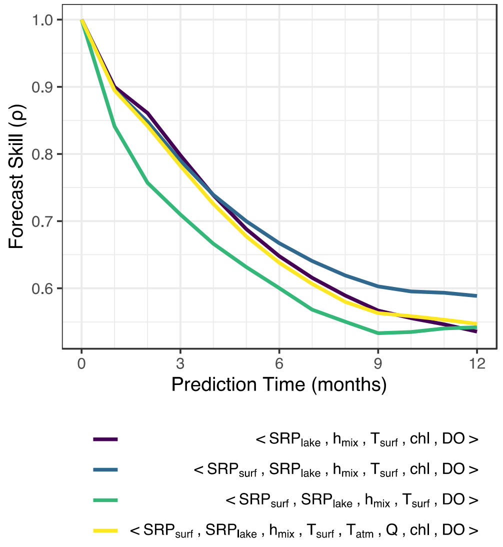

Figure S6: Identifying robust set of drivers for explicit ("mechanistic") empirical dynamic model for predicting month-to-month changes in DO_B when previous DO_B is included as an EDM variable. Near-term (1-month) forecast skill of DO_B is high due to fundamental autocorrelation in the time-series, which makes distinguishing between embeddings difficult once DO_B itself is included as a variable (in contrast to Fig 1D). If we apply iterative forecasting with S-map, however, difference in skill becomes more apparent. For DO_B, we find that eliminating the exogenous physical variables of Rhone discharge (Q) and atmospheric temperature (T_{atm}) leads to more robust forecast skill through time, while eliminating any of the biogeochemical variables leads to decreased performance. Consequently, we focus S-map coefficient and hybrid modelling efforts on the embedding $TP_{lake}(t)$, $TP_{surf}(t)$, $h_{mix}(t)$,$T_{surf}(t)$, CHL(t), $DO_B(t)$ to predict changes in DO_B through time.

SI References

1. R. Schwefel, A. Gaudard, A. Wüest, D. Bouffard, Effects of climate change on deep-water oxygen and winter mixing in a deep lake (Lake Geneva): Comparing observational findings and modeling. *Water Resour. Res.* **52**, 8811–8826 (2016).
2. R. Gächter, B. Müller, Why the phosphorus retention of lakes does not necessarily depend on the oxygen supply to their sediment surface. *Limnol Oceanogr* **48**, 929–933 (2003).
3. A. M. Michalak, *et al.*, Record-setting algal bloom in Lake Erie caused by agricultural and meteorological trends consistent with expected future conditions. *Proc Natl Acad Sci U S A* **110**, 6448–6452 (2013).
4. R. A. Vollenweider, The scientific basis of lake and stream eutrophication, with particular reference to phosphorus and nitrogen as eutrophication factors. *Organisation for Economic Cooperation and Development, Paris* **159** (1968).
5. F. Pomati, B. Matthews, J. Jokela, A. Schildknecht, B. W. Ibelings, Effects of re-oligotrophication and climate warming on plankton richness and community stability in a deep mesotrophic lake. *Oikos* **121**, 1317–1327 (2012).
6. D. Finger, A. Wüest, P. Bossard, Effects of oligotrophication on primary production in peri-alpine lakes. *Water resources research* **49**, 4700–4710 (2013).
7. E. Jeppesen, J. P. Jensen, M. Søndergaard, Response of phytoplankton, zooplankton, and fish to re-oligotrophication: an 11 year study of 23 Danish lakes. *Aquat Ecosyst Health Manag* **5**, 31–43 (2002).
8. D. Gerdeaux, O. Anneville, D. Hefti, Fishery changes during re-oligotrophication in 11 peri-alpine Swiss and French lakes over the past 30 years. *Acta oecologica* **30**, 161–167 (2006).
9. O. Anneville, *et al.*, The paradox of re-oligotrophication: the role of bottom–up versus top–down controls on the phytoplankton community. *Oikos* **128**, 1666–1677 (2019).

10. H. W. Paerl, J. Huisman, Blooms like it hot. *Science* **320**, 57–58 (2008).

11. R. I. Woolway, C. J. Merchant, Worldwide alteration of lake mixing regimes in response to climate change. *Nat. Geosci.* **12**, 271–276 (2019)

12. M. Hondzo, Dissolved oxygen transfer at the sediment-water interface in a turbulent flow. *Water Resour. Res.* **34**, 3525–3533 (1998).

13. G. Sugihara, *et al.*, Detecting causality in complex ecosystems. *Science* **338**, 496–500 (2012).

14. V. Berthon, *et al.*, Trophic history of French sub-Alpine lakes over the last similar to 150 years: phosphorus reconstruction and assessment of taphonomic biases. *Annales de Limnologie - International Journal of Limnology* **72**, 417 (2013).

15. O. Anneville, J. P. Pelletier, Recovery of Lake Geneva from eutrophication: quantitative response of phytoplankton. *Archiv für hydrobiologie* **148**, 607–624 (2000).

16. N. Salmaso, Life strategies, dominance patterns and mechanisms promoting species coexistence in phytoplankton communities along complex environmental gradients. *Hydrobiologia* **502**, 13–36 (2003).

17. N. Salmaso, Ecological patterns of phytoplankton assemblages in Lake Garda: seasonal, spatial and historical features. *J Limnol* **61**, 95 (2002).

18. K. Tapolczai, *et al.*, Occurrence and mass development of Mougeotia spp. (Zygnemataceae) in large, deep lakes. *Hydrobiologia* **745**, 17–29 (2015).

19. B. Alric, *et al.*, Local forcings affect lake zooplankton vulnerability and response to climate warming. *Ecology* **94**, 2767–2780 (2013).

20. O. Anneville, C. Vogel, J. Lobry, J. Guillard, Fish communities in the Anthropocene: detecting drivers of changes in the deep peri-alpine Lake Geneva. *Inland Waters* **7**, 65–76 (2017).

21. B. Müller, T. Steinsberger, A. Stöckli, A. Wüest, Increasing Carbon-to-Phosphorus Ratio (C:P) from Seston as a Prime Indicator for the Initiation of Lake Reoligotrophication.

Environ. Sci. Technol. **55**, 6459–6466 (2021).

22. H. Ye, G. Sugihara, Information leverage in interconnected ecosystems: Overcoming the curse of dimensionality, *Science* **353**, 922-5 (2016).
